# Molecular characterization of a novel cryptic virus infecting pigeonpea plants

**DOI:** 10.1371/journal.pone.0181829

**Published:** 2017-08-03

**Authors:** Surender Kumar, Burra L. Subbarao, Reenu Kumari, Vipin Hallan

**Affiliations:** 1 Academy of Scientific and Innovative Research (AcSIR), CSIR-Institute of Himalayan Bioresource Technology (CSIR-IHBT) Campus, Palampur, India; 2 Department of Biotechnology, Plant Virus Lab, CSIR-Institute of Himalayan Bioresource Technology, Palampur, Himachal Pradesh, India; 3 Sainikpuri, Secunderabad, Telangana state, India; National Botanical Research Institute CSIR, INDIA

## Abstract

A new member of the genus *Deltapartitivirus* was identified containing three dsRNAs with an estimated size of 1.71, 1.49 and 1.43 kb. The dsRNAs were extracted from symptomless pigeonpea [*Cajanus cajan* (L.) Millspaugh] plants cv. Erra Kandulu. This new virus with 4.64 kb genome was tentatively named Arhar cryptic virus-1 (ArCV-1). The genomic RNAs were amplified and characterized by sequence independent single primer amplification. The dsRNAs shared a highly conserved 16 nt 5’ non-coding region (5’-GATAATGATCCAAGGA-3’). The largest dsRNA (dsRNA-1) was identified as the viral RNA dependent RNA polymerase (replicase), predicted to encode a putative 55.34 kDa protein (P1). The two other smaller dsRNAs (dsRNA-2 and dsRNA-3) predicted to encode for putative capsid proteins of 38.50kDa (P2) and 38.51kDa (P3), respectively. Phylogenetic analysis indicated that ArCV-1 formed a clade together with Fragaria chiloensis cryptic virus, Rosa multiflora cryptic virus and Rose cryptic virus-1, indicating that ArCV-1 could be a new member of the genus *Deltapartitivirus*. ArCV-1 3D^pol^ structure revealed several interesting features. The 3D^pol^ in its full-length shares structural similarities with members of the family *Caliciviridae*and family *Picornaviridae*. In addition, fourth dsRNA molecule (dsRNA-2A), not related to ArCV-1 genome, was found in the same plant tissue. The dsRNA-2A (1.6 kb) encodes a protein (P4), with a predicted size of 44.5 kDa. P4 shares similarity with coat protein genes of several cryptic viruses, in particular the bipartite cryptic viruses including Raphanus sativus cryptic virus-3. This is the first report of occurrence of a cryptic virus in pigeonpea plants.

## Introduction

Pigeonpea [*Cajanus cajan* (L.) Millspaugh], having originated in India, is one of the major grain legume (pulse) crops in the Indian subcontinent. The crop is known to be susceptible to a few virus and virus-like pathogens, belonging to different genera [1–4]. While characterizing the dsRNA from the sterility mosaic disease (SMD) [3] affected pigeonpea plants, we noticed that two sets of symptomless controls plants containing four dsRNA molecules while none were noticed in the remaining sets of healthy samples. Healthy control plant samples (Mg1-H1 and Mg1-H2) that contained the dsRNA were collected from one of the farmers’ fields in Chevella area (near Hyderabad, Telangana state). The pattern and the number of dsRNA segments did not match with dsRNAs associated with pigeonpea plants infected by PPSMV-P sub isolate-Chevella (separate manuscript under preparation). Sequence of the four dsRNAs showed neither homology nor alignment to any region of PPSMVs. However, these dsRNAs share close similarities with individual genomic segments of several plant cryptoviruses. Evidence is provided to show that three of the four dsRNAs constitute the genome of a new cryptovirus tentatively named “Arhar cryptic virus-1” (ArCV-1) with a genome size of 4.64 kb. Pigeonpea is referred to as “Arhar”in Hindi. The genomic sequences showed its phylogenetic relationship to members of the genus *Delatapartitivirus*. The fourth dsRNA (dsRNA-2A) contained cryptic virus coat protein- like sequences with no similarity to that of ArCV-1 genome. We discussed different possibilities of its occurrence with ArCV-1 genome and its relevance.

Cryptic viruses usually contain 2 to 3 monocistronic genomic dsRNA segments which are encapsulated individually [5, 6], and referred to as bipartite and tripartite viruses. Occurrence of tripartite cryptoviruses is less frequent [7]. Plant cryptoviruses, in general are either pollen or seed transmitted, belong to the family *Partitiviridae* which include five genera viz. *Alphapartitivirus*; *Betapartitivirus*; *Gammapartitivirus*; *Deltapartitivirus* and *Cryspovirus* [8]. Members of the family *Partitiviridae* are known to infect mainly fungi, plants and protozoans. This is the first report detailing association of a cryptic virus with pigeonpea (*Cajanus cajan*(L.) Millspaugh) plants which belongs to the family *Fabaceae*, harboring one third of the reported cryptovirus infections.

Mixed infections involving different cryptoviruses as well as host specific pathogenic viruses are common [5, 9, 10], effecting a single plant or more. Beet cryptic virus- 1 (BCV-1) and beet cryptic virus- 2 (BCV-2) were reported infecting the same host [9], as a mixed infection, similarly in the case of pepper where Pepper cryptic virus-1 (PepCV-1) and Pepper cryptic virus-2 (PepCV-2) were found as mixed infection [11].

In the present study, ArCV-1 genomic segments were characterized by Sequence Independent Single Primer Amplification (SISPA) method. This comprehensive method was found to be precise with a high degree of specificity to investigate the evolution and genetic diversity in dsRNA viruses. The four dsRNAs eluted from the agarose gel were purified and have been used as templates for RT-PCR amplification employed in SISPA to generate full-length cDNAs.

It is of interest to examine if ArCV-1 RNA dependent RNA polymerase (RdRp) structurally resembles the known RdRp of the dsRNA bacteriophage Փ-6, reovirus, or with other viruses like calciviruses and picornaviruses [12–16]. Their RdRp molecules, whose structural details have been described, are larger than RdRp of ArCV-1. RdRps of several important human and animal picornaviruses have been extensively studied that correlated functions with structural details, which contributed to the understanding of the mechanics of this enzyme activity leading to viral replication [12–16]. Similar efforts were made in the past decade to study the 3D structural characterization of RdRps of a very few plant viruses and detailed studies remained to be carried out for RdRps of double-stranded plant viruses including partitiviruses. ArCV-1 RdRp sequence analysis revealed the presence of several conserved amino acid sequence motifs common in other tripartite cryptoviruses. These motifs have been described to be important for biological functions in several RdRps [17]. We report here the results of elaborated computer-assisted analysis of ArCV-1 replicase which revealed the presence of conserved sequence motifs (A to G) present in the fingers and palm subdomains of the polymerase that are shared in most of the RdRps. Interestingly, ArCV-1 replicase has more structural resemblances with several members of ssRNA (+) mono-partite Picornaviruses (viral replication by primer-dependent initiation), than the *de novo* dsRNA bacteriophage Փ-6 and reovirus polymerases. Variations found in ArCV-1 motifs’ sequence that may be involved in polymerization mechanism and the conserved motifs unique to cryptoviruses have been described. This report illustrates several interesting features of the ArCV-1 3D^pol^ and its complete structure was determined.

## Results

### Isolation of dsRNAs from pigeonpea plants and their amplification by SISPA

**Fig 1:**
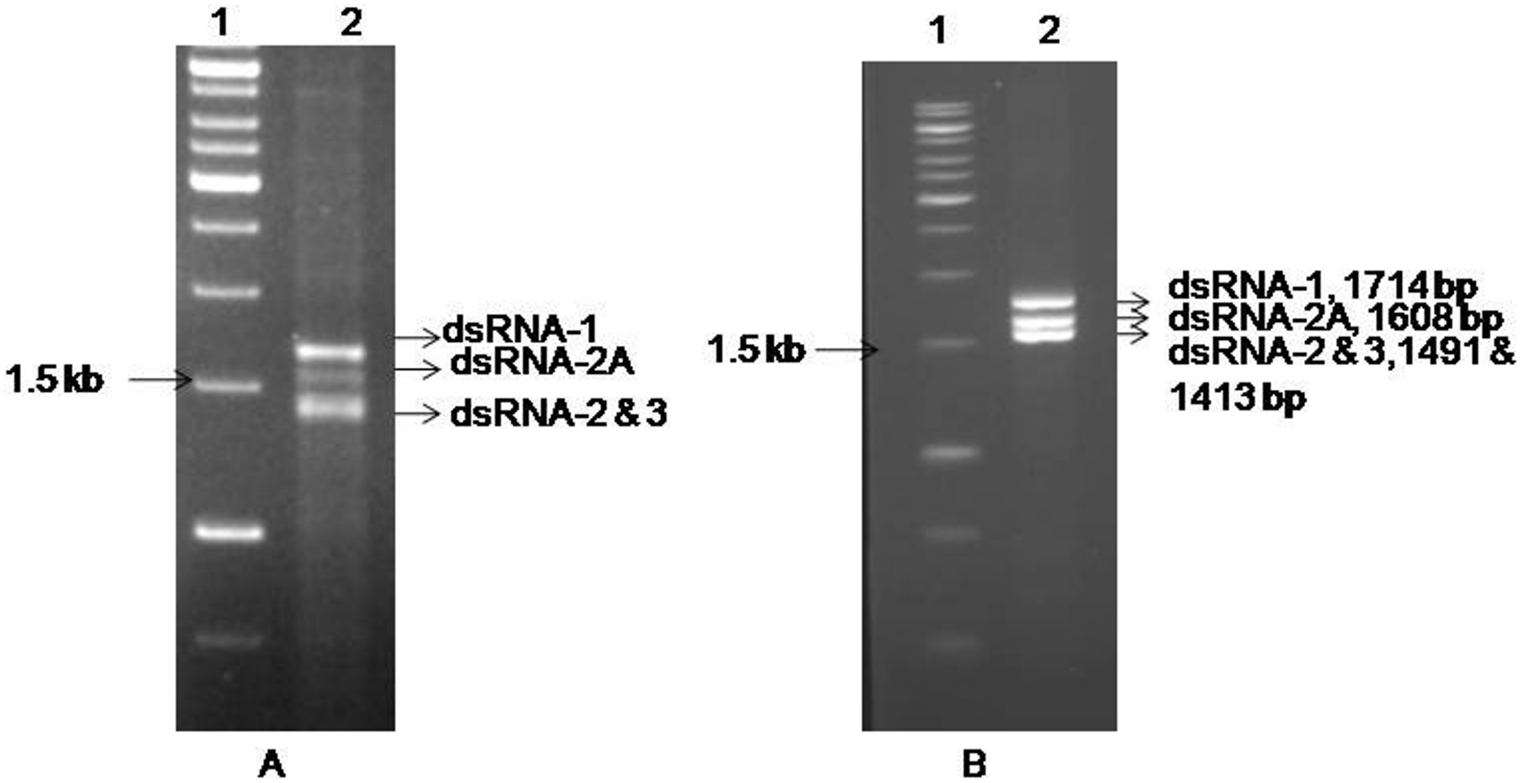
Resolution of dsRNA species isolated from symptom free pigeonpea plants and SISPA-PCR amplified dsRNAs fractionated on 1.5% agarose gel. **(A)** Lane-2, dsRNAs isolated from symptomless pigeonpea field collected plants; **(B)** Lane-2, amplified dsRNAs. The ArCV-1, dsRNA-2 and 3 were almost similar in size. Lane 1 (A, B) contains 1kb DNA marker (Fermentas, Thermo scientific, USA).

**Fig 2:**
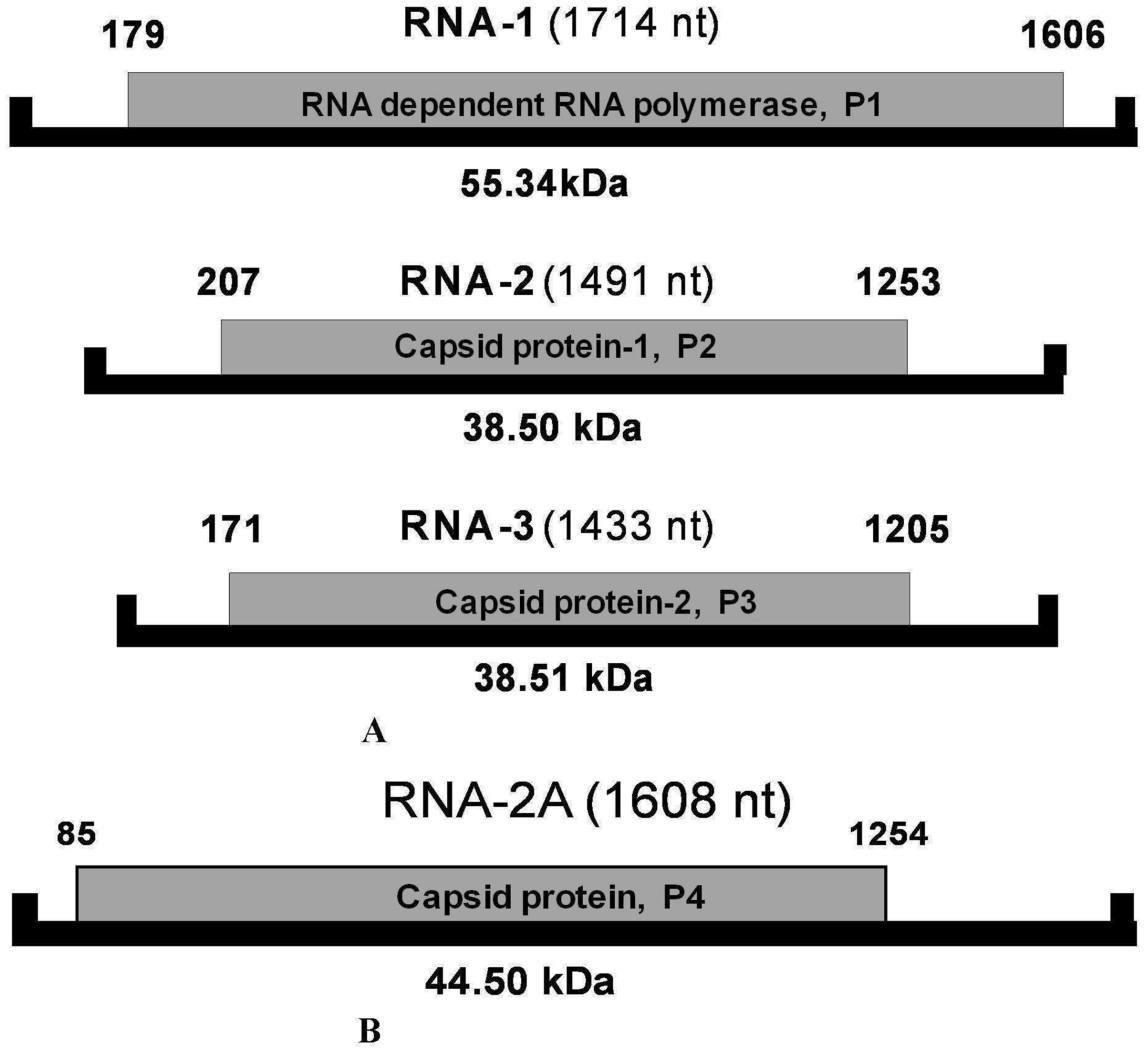
Schematic representation of genome organization of Arhar cryptic virus-1(ArCV-1) and associated CP-like dsRNA-2A. **(A)** ArCV-1, dsRNAs were denoted (RNA1, RNA2 and RNA3) are represented by solid black lines. The larger RNA was identified putative RdRp encoding a single peptide of P1, marked as grey box. The two smaller RNAs encode two individual peptides of P2 and P3 and were identified as putative capsid proteins, marked as grey boxes. **(B)** Schematic representation ofa fourth dsRNA isolated along with the ArCV-1 genome with an unusually long 3’NTR, referred as RNA-2A (Black solid line) is predicted to encode a putative coat protein P4 marked as grey box.

Symptomless pigeonpea leaf samples, Mg-H1, Mg-H2 collected from MG-1and MG-2 fields contain four dsRNA species with an estimated size of ~1.71, ~1.49, ~1.43 and ~1.6 kb (Figs 1A and 2), whereas as symptomless leaf sample (Mg-H3) collected from another field (MG-3) were devoid of similar dsRNAs. The pure dsRNAs yields ranged 700 to 800 ng was obtained from 7g of fresh leaves with consistent dsRNA unique profile which is entirely different from the PPSMVs. Post SISPA, PCR amplification product profile was similar to the dsRNAs obtained from the initial dsRNA extraction from the symptomless leaves (Fig 1). These amplification products were cloned and characterized.

### Genome organization of ArCV-1

**Fig 3:**
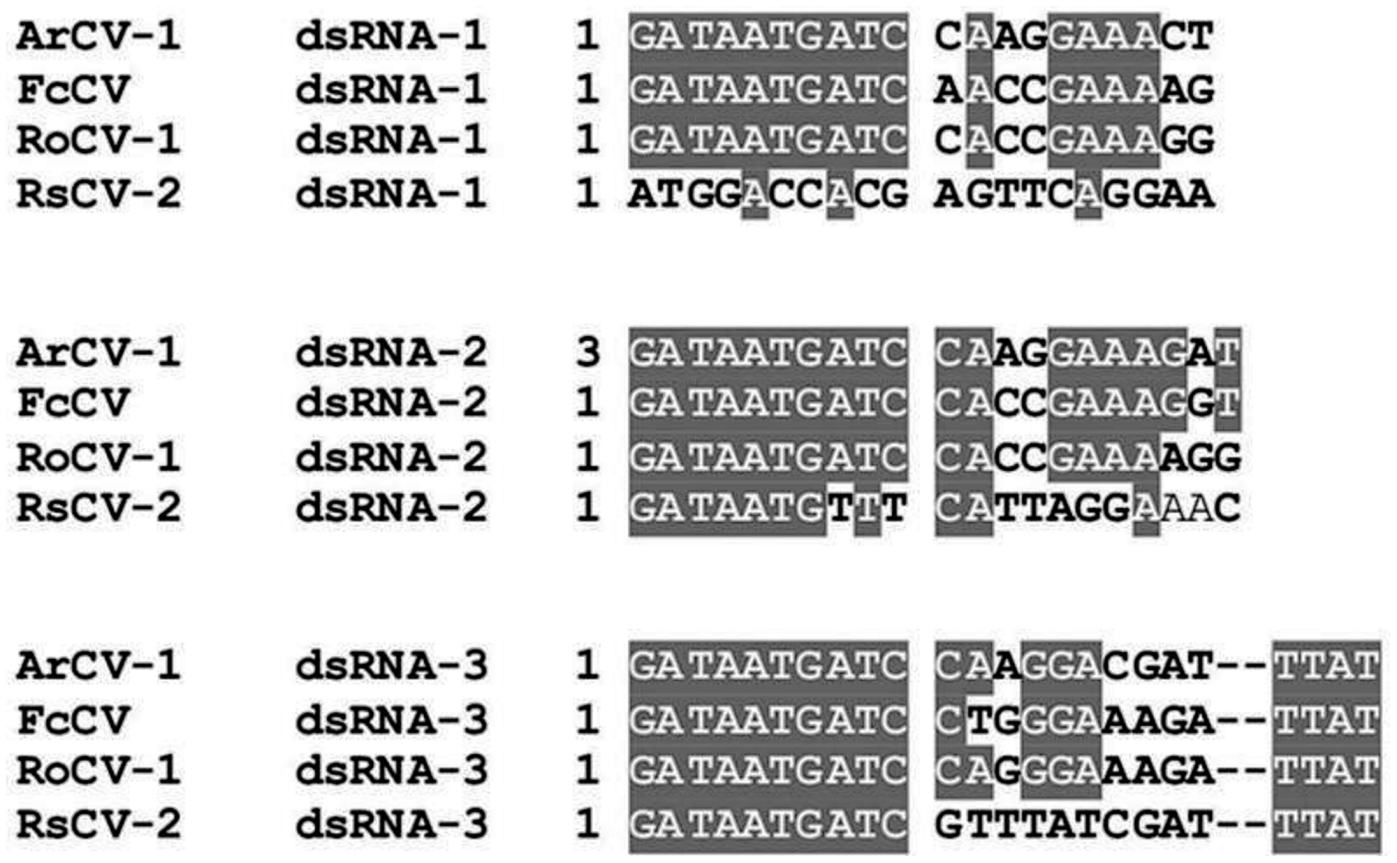
Alignment of the nucleotide sequences of 5’ non-coding regions of dsRNAs-1, 2 and 3 of ArCV-1, Fragaria chiloensis cryptic virus (FcCV), Rose cryptic virus-1 (RoCV-1) and Raphanus sativus cryptic virus-2 (RsCV-2). Conserved residues are marked as in grey box.

The cloned amplification products corresponding to the four dsRNAs were sequenced. BLAST analyses of the full-length sequences revealed their identity with the orthologs of several cryptoviruses. ArCV-1 genomic segments shared distinctive conserved 16 nt stretch (GATAATGATCCAAGGA) at the 5’- non-translated region (NTR), a feature commonly observed in multipartite cryptic viruses and constituted as the genome of ArCV-1 with a size of 4.64 kb (Fig 3). Whereas the dsRNA-2A, 5’-terminus sequence (AGAATTTGCCCTGTAT) did not share the conserved sequences and thus treated as a separate entity. The genome organization of ArCV-1 was thus established during the present study (Fig 2), together with dsRNA-2A depicting the single open reading frames (ORFs) of dsRNA-1 (RdRp), dsRNA-2 (CP-1) and dsRNA-3 (CP-2) with the predicted molecular weights of proteins encoded by the RNAs (Table 1). Unlike the 5’ end, the 3’-region is least conserved in several cryptic viruses. The majority of the members of the family *Partitiviridae* typically contain few pyrimidine bases conserved at the 3’-terminal and in tripartite viruses, conservation is seen mainly in two of the RNA segments. ArCV-1 RNAs encoding capsid proteins are conserved in the last three nt (TTC), like in Raphanus sativus cryptic virus-2 (RsCV-2), the three ArCV-1 RNAs end with TC. The majority of the Deltapartitiviruses contain RNA segments consistently ending with nucleotides TC except in Rosa multiflora cryptic virus (RmCV) and Cannabis cryptic virus (CanCV) (*Betapartitivirus*) interestingly, the 3’ termini, each with a poly (A) tract.

**Table 1:** Genome characteristics of aArCV-1, encoded proteins and associated dsRNA-2A.

ArCV-1 genomic segments, dsRNA-2A and putative function	5’UTR	Coding region	
dsRNA-1 (HG797710); RdRp; 1,714 nt long, 475 aa, 55.34 kDa	108 nt	1,606 nt-179 nt	178 nt
dsRNA-2 (HG967639); CP-1; 1,491 nt long, 348 aa, 38.5 kDa	206 nt	1,253 nt-207 nt	237 nt
dsRNA-3 (HG967638), CP-2; 1,433 nt long, 344 aa, 38.51 kDa	170 nt	1,205 nt-171 nt	227 nt
dsRNA-2A (HG917912), P4; CP -like; 1,608 nt long, 389 aa, 44.5 kDa	84 nt	1,608 nt-85 nt	353 nt

The dsRNA-1 (HG797710) is 1,714 nt long, containing a single open reading frame (ORF) between nt 1,606 and nt 179 with 178- and 108-nt- long 5’ and 3’ NTRs, respectively (Fig 2, Table 1). This ORF encodes for a 55.34 kDa protein (P1) containing 475 amino acids. P1 protein was identified as viral RdRp and contains conserved short amino acid sequence motifs (A to G) which are essential for viral replication [18–21], and known in several picornavirus RdRPs. The A–G motifs are conserved in the RdRps of several genera of plant and animal viruses containing monopartite, linear, ssRNA (+) genome [17, 18, 22], segmented, tripartite ssRNA (+) viruses [23], as well as multi-segmented linear ssRNA (-) viruses [24–27]. ArCV-1 RdRp similar to the other cryptoviruses contained these signature motifs in the central region of the polypeptide. Specific amino acid sequences mostly conserved formed these seven motifs: S**S**A**AGYG**YVGLK (motif G; 117–128) **K**V**R**NV**W**//DP**L**//SF**Y**FI**G**Q**D** (motif F; 181–219), F**D**WSGFD (motif A; 241–247), GIP**S**GS**C**F**T**N**I**IGSITN (motif B; 302–318), THG**DD** (motif C; 338–342), D**K**SD (motif D; 372–375), and TF**L** (motif E; 384–386) known to be functionally active in viral replication [17]. Computer analysis predicted the active residues in each of the conserved motives (S1 and S2A Figs), indicated in bold letters. Motif G is less conserved amongst the human and animal viruses belonging to the picornavirus ssRNA (+) super family as well as viruses containing dsRNA genome. Active residues in motif G are comparatively well conserved in the dsRNA cryptovirus RdRps (S1 Fig). Aided by RdRp sequence of Փ-6 and reovirus active residues in motif G were identified [12, 13]. Conserved proline (in this motif) of most of the picornaviruses is substituted in ArCV-1 RdRp by glycine in the C-terminus. SSAAGYGY-G-K sequence is highly conserved in cryptic viruses. In addition to the above mentioned seven conserved motifs, several conserved sequences in ArCV-1 RdRp were present flanking the central region towards 5-’ and 3’-terminal regions. Motifs GWARS (53–57), STPD (162–165), TRTQL (168–172) are present in N-terminus and RDE (397–399), CLRML (402–406), LRA (421–423), DAG (429–431) YLY (436–438), WDP (460–462) in the C-terminus devoid of predicted active residues. Motifs GWARS and TRTQL (motifs-1and 2, S1 Fig) were conserved in most of the bi- and tripartite cryptoviruses. However, specific functions of these exclusive motifs are yet to be determined.

**Fig 4:**
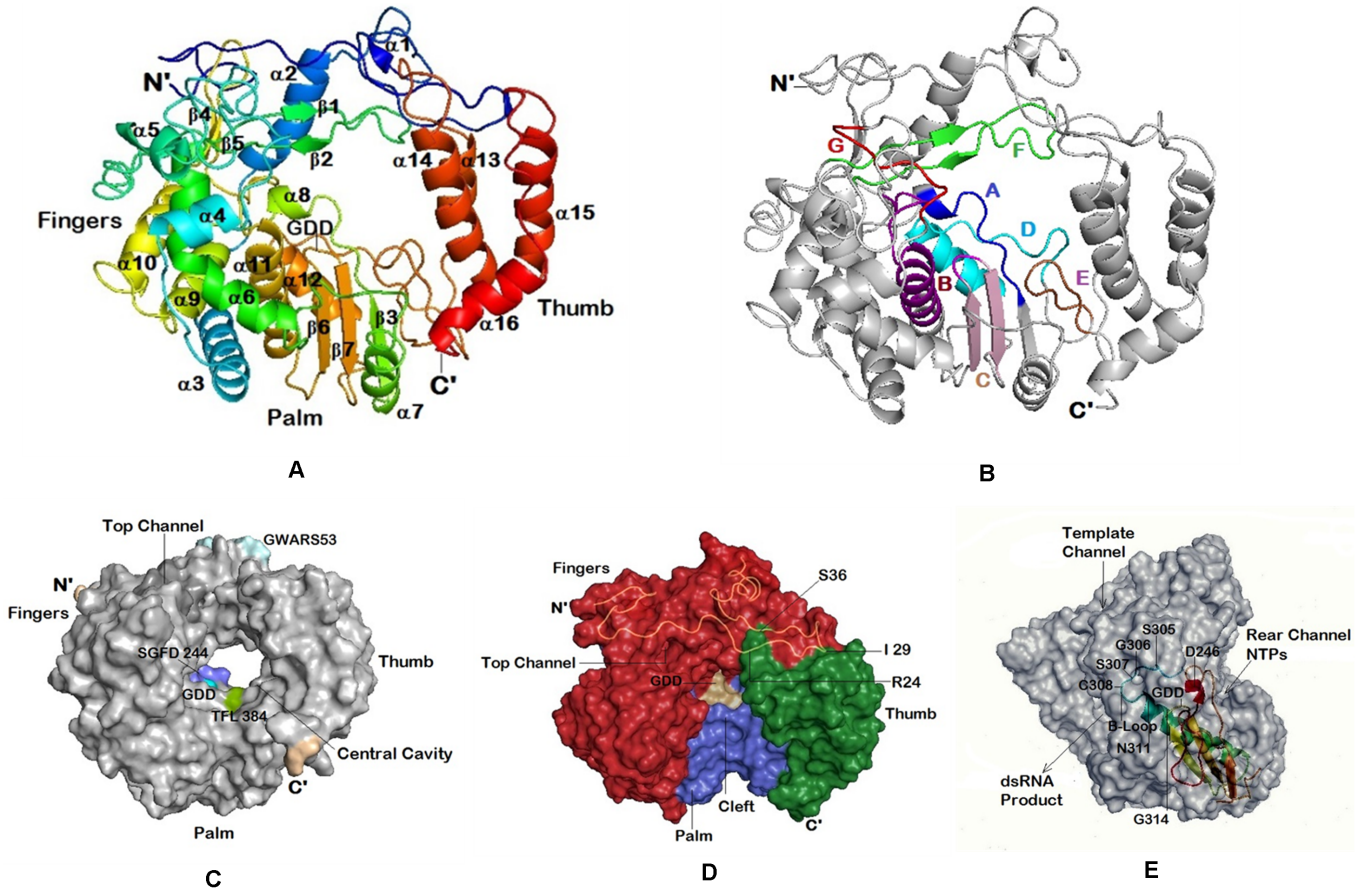
Molecular graphics of ArCV-1 RdRp 3D structure at 7.9Å resolution. Molecular graphic of ArCV-1, RdRp 3D structure of 7.9Å resolution with central cavity flanked by N-terminus and C-terminus structures and the folding topology resemble the RdRps of picornaviruses: **(A)** Cartoon representation of the molecule in a conventional orientation of the closed right hand depicted with sub-domains, fingers (shades of blue- cyan), thumb (shades of red) and palm. **(B)** Cartoon representations of ArCV-1 3Dpol Motifs A, B, C, D, E and F are represented in blue, purple, light pink, cyan, brown and green, respectively. (**C**) Surface representation of the ArCV-1 3D polymerase showing a donut shaped structure with a central catalytic cavity and the top channel. Few of the representative motifs of ArCV-1 3D with residues known to interact with the RNA and the incoming NTP substrate were identified close to the catalytic center (GDD cyan). (**D**) ArCV-1 3D^pol^, turned 90° to the front showing view from the top, with three subdomains, Fingers, Thumb and Palm farming putative RNA binding cleft. The N-terminal (1–55 residues) over layered on the enzyme surface to show the fingers region tether to the thumb region (shown as yellow coil with residues Arg-24, I-29, S-36 binding to the thumb subdomain). **(E)** Structural details of the altered conformation of motif A and B side view of the RdRp surface representation exposing the three channelsArCV-1 polymerase that are described for the picornoviruses. Positioning of channels as template channel (top) NTPs channel (rear) and product exiting channel (front) were denoted. The subdomain motifs A, B, and C in part, as cartoon was over layered to exact position. Figures were developed with PyMol.

Motif C represented by GDD sequence (Fig 4D, S2A Fig) is almost universal and has been described as the center of catalytic activity of the RdRp in all RdRp classes [18, 28]. The 3D structural analysis revealed that the GDD sequence present in the loop formed by antiparallel β–sheets 6 and 7 is located in the bottom part of the central cavity of the molecule in the palm region (Fig 4B, S2A Fig). Several investigators conducted mutational studies of the catalytically important residues of the polymerase of different viruses [29–32], and determined that the first aspartate is an essential residue of this highly conserved motif for the enzyme activity and its coordination in the binding of a magnesium ion that would be part of the rNTP binding site [12, 33]. It was found that the first Asp (D341 in ArCV-1) is a strict requirement and any change leads to loss of the enzyme activity. Some flexibility was suggested with regard to glycine and the second aspartate and subtle changes in the position of these two residues are tolerated with exceptions [34, 35]. Similar observations were made when the conserved aspartate of brome mosaic virus (+ssRNA) was replaced in the 2a gene with glutamate (D470E), as the DR4 mutant did not replicate in the barley protoplasts [36]. The polymerase structural domains and the regulatory mechanism of the enzyme with respective different viruses are well documented in a recent review [17]. Possible functions of the residues of the A to G motifs described for identical RdRps was conserved with respect to the ArCV-1 3Dpol structure and was discussed in structural analysis of ArCV-1 RdRp section. ArCV-1 RdRp has a high degree of homology with RdRps of tripartite viruses belonging to the genus *Deltapartitivirus*, compared to the RdRps of bipartite viruses. The RdRp has 72.4% amino acid sequence identity with FcCV P1, 71.2% with RmCV P1 and percent sequence identity of 71.0 to 12.1% with the P1 proteins of other tripartite viruses (S1 Fig, Table 2) as compared to 35–39% sequence identity found with RdRps of bipartite viruses. The 3’-terminus contained the sequence GCACCCGTCTC.

**Table 2:** Percent sequence identity of dsRNA genomic segments of ArCV -1 with tripartite and bipartite cryptic viruses.

Cryptovirus	ArCV-1		
	dsRNA-1	dsRNA-2	dsRNA-3
**Tripartite viruses**			
FcCV	72.4	39.1	40.1
RmCV	71.2	17.2	19.9
RoCV-1	**71.0**	**40.6**	**42.7**
RsCV-2	59.0	28.9	26.4
AoV	16.9	8.91	9.40
RsCV-1	14.8	12.6	13.1
BCV-2	12.1	8.33	9.0
**Bipartite viruses**			
*PerCV	38.8	9.6	--
RsCV-3	38.4	9.3	--
PepCV-2	37.9	10.6	--
FCV	35.2	12.3	--

*PerCV: Persimmon cryptic virus

The dsRNA-2 (HG967639) is 1,491nt long, with a single ORF (nt 207 to 1253) of a 38.5 kDa protein with 348 amino acids (P2). This RNA contains 206- and 237-nt as the 5’and 3’NTRs, respectively (Fig 2, Table 1) and the 3’ terminus contained the sequence “GCA CCCATATTC”. The P2 was identified as the capsid protein-1, having 40.6% amino acid sequence identity with corresponding protein of RsCV-1- (EU024677) (Table 2).

The dsRNA-3 (HG967638) is 1,433 nt long, containing a single ORF between nt 171 and 1,205 with 170- and 227nt long 5’ and 3’ NTRs, respectively (Fig 2, Table 1) and the 3’ terminus contained the sequence “GCACCACGTTTC”. This ORF encodes a 38.51 kDa protein (P3) of 344 amino acids, identified as the second capsid protein. The P3 protein has 42.7% sequence identity with coat protein (RNA-3) of RsCV-1 (EU024677) (Table 2).

The dsRNA-2A (HG917912), extracted along with the ArCV-1 genome was found in lower concentration in comparison to the genomic dsRNAs (Fig 1A). The RNA is 1,608 nt long, contains a single ORF that encodes a 44.5 kDa protein (P4) of 389 amino acids. The ORF starts at nt. 85 and terminates at 1,254 nt (Fig 2B, Table 1) with 84- and 353-nts as the 5’ and unusually long 3’- NTRs, respectively. Its 5’ terminus has AGAATTTGCCCTGTAT sequence and did not share sequence identity neither with the corresponding region of ArCV-1 genomic segments (Fig 3), nor with the C-terminus (TTC) of the capsid proteins.

Amino acid sequence identity analysis of P4 revealed similarity with the putative genomic capsid proteins of several cryptic viruses (Table 3), as well as few eukaryotic genes of plant genomes. P4 shares 36.7% sequence identity s with coat protein of RsCV-3 (FJ461350), 35.8% with Citrullus lanatus cryptic virus (CiLCV; KC429583), 34.4% with PepCV-1 (JN117277) and 26.0% PepCV-2 (JN117279) (Table 3). Interestingly, the P4 protein has high degree of sequence identity with cryptic virus coat proteins of bipartite viruses (Table 3). Efforts were made to confirm the presence of a second RdRp assuming there may be in existence and dsRNA-2A belongs to another cryptic virus, but were not successful. In each attempt similar four dsRNAs were isolated. It is hard to explain, in absence of a related RdRp gene, and to speculate that the dsRNA-2A coat protein like sequence belong to a yet unknown second cryptovirus infecting pigeonpea.

**Table 3:** Percent sequence identity of amino acids of P4 protein (dsRNA-2A; HG917912) shared with cryptic virus coat proteins.

Bipartite Cryptovirus			Tripartite Cryptovirus		
Cryptovirus	Acc. No.	Identity	Cryptovirus	Acc. No.	Identity
RsCV-3	FJ461350	36.7	BCV-2 CP2*	HM560704	27.94
CiLCV	KC429583	35.8	RsCV-1 CP1	DQ181926	12.06
PepCV-1	JN117277	34.4	AoV CP1	EU118278	11.52
PerCV	HE805114	29.1	FcCV CP2	DQ355439	07.78
PepCV-2	JN117279	26.0	RoCV-1 CP2	EU413668	07.00

* Coat protein species that has maximum sequence identity of a tripartite cryptovirus was considered

Apart from this, the four RNA showed significant sequence homology with plant genes. Interestingly in BlastX, RNA-1 showed 64% sequence identity with unknown protein of *Picea sitchensis* or Sitka spruce (ADE76327), an evergreen conifer tree. RNA-2 showed 34% sequence identity with unknown protein (XP008459635) of *Cucumis melo*. Further, RNA-3 showed highest sequence identity with plant genes as 61% with uncharacterized proteins KOM46435 and XP017431256 of *Vigna angularis*. Similarly, dsRNA-2A shared 34% sequence identity with ATP-binding cassette (ABC) transporter F family member-1 (KHF98106) of *Gossypium arboretum* (tree cotton) found conserved in eukaryotes and prokaryotes. These eukaryotic lineages are not the known host plants for cryptic viruses.

### Structural analysis of the ArCV-1 RdRp

The peptide sequence of ArCV-1 RdRp (P1) was used for developing the 3Dpol structure. Structural templates were identified from the PDB and the 3D model created using Zhang lab server [37, 38]. PyMol was used to develop the three dimensional figures. Crystal structure of RdRp has been described depicting the “closed right hand” configuration [17, 39]. RdRps belonging to various genera of viruses containing amino acid sequence motifs are most conserved and occur in a typical order [20, 28, 40], reflecting structural conservation in most cases. Besides size, origin, and some structural variations, all classes of RdRp including several known picornaviruses share two main features by (1) having the 3D structure divided into three major subdomains described as palm, thumb and fingers [17, 39, 41, 42], and (2) the interconnected subdomains that encloses the conserved catalytic region (Fig 4, S2 Fig) within a largely hollow center referred to as the catalytic center [43]. The 55 kDa ArCV-1 3D^pol^ is a spherical molecule resolved at 7.9Å resolution showed the basic features described above comprising of characteristic 16 α-helices, 7 β-sheets interconnecting with coiled structures (Fig 4A) forming a structure of a cupped right hand. Unlike in picornaviruses the partitivirus genome is devoid of 5’ terminus genome-linked protein (Vpg) or particularly the polymerase with polyadenylated C’- terminal with the exception of CanCV (JN196536) and RsCV-1RNA-3 (DQ181927).

The N-terminal residues (1–55) in fingers subdomain (Fig 4A, Blue) of the RdRp molecule extend over to the thumb subdomain binding them together (Fig 4A and 4D) encircling the molecule, ensuing central cavity. This architecture makes the molecule spherical or globular (Fig 4A and 4C), and not U shape normally seen in DNA polymerases and DNA-dependent RNA polymerases [44]. ArCV-1 3D pol has a big cleft opening in to central cavity groove in the front and one at the top left channel referred to as template channel leads to the centre of the cavity supposed to be allowing the access of the RNA template and NTP substrates to the central cavity [17, 33, 45, 46] and that central cavity opens out to the side on the right (rear channel) (Fig 4E).

The N-terminal portion of the fingers subdomain is mainly composed of random coiled web (residues 1–55) on the top portion molecule sometimes referred as index finger with the N-terminal (Met 1) protruding out. N-terminal coiled structure runs across, connecting the fingers and the thumb subdomains, folds back on itself into a loop providing the conserved residues GWARS (53–57) seem to be unique to the bi- and tripartite cryptoviruses (S2A Fig) located at the top of the molecule.

The fingers subdomain (residues 56–232 and 252–309) consist of eight α-helices (α2 to α7 and α9, α10) and four β-strands (β1—β2 and β4-β5). Broad structural variations are seen in fingers subdomain followed by the thumb in general than the palm in the viruses belonging to picornaviruses. Whereas these variations in fingers subdomain being less significant in cryptic viruses. The fingers region contains motif F and motif G, the latter being geometrically closer to the catalytic center of the palm subdomain.

**Motif F:** A stretch of 42 amino acid residues mostly conserved in cryptoviruses spanning few residues before β-sheet 2 to α-6 and connecting coil containing conserved active residues as motif F. Like the N-terminal long loop, a portion of motif F extends from the fingers to interact with the thumb. The size of the loop, however, varies in different RdRps [21]. Motif F contains predominantly hydrophobic residues, along with at least six active residues (R183, W186, L203, Y214, G217 and D219) supposed to be interacting with the RNA in the ArCV-1 3D complex (S1 Fig) along with the conserved Lys181. Basic residues Lys181 and Arg183 of ArCV-1 3D RdRp have been identified associated with motif F of other RdRps; FMDV (K172). Lys172 residue along with two arginine residues (R168 and R179) predicted to interact with the incoming NTP substrate in FMDV RdRp [33]. Motif F of cryptoviruses unlike the other classes of RdRps is well conserved.

**Motif G:** The motif consists of residues from amino acid positions 117 to 128 (S**S**A**AG**Y**G**YVGL**K**). This motif is highly conserved in cryptoviral RdRps. Amino acid residues of motif G form part of a long coiled loop connecting α-helices 4 and 5 in the fingers subdomain (Fig 4B) of the 3D pol. Motif G is not well conserved in general and does not exist in Human immunodeficiency virus-1 (HIV-1) RT, but observed across several genera of plant, animal and human picornaviruses, as noted from RdRp sequences [47]. Glycine and lysine seem to be a strict requirement of motif G as they are conserved (Gly121 and Lys128 in ArCV-1) in all classes of RdRp. The conserved proline residue noticed in some picornavirus [Avian infectious bronchitis virus (AIBV), Severe acute respiratory syndrome-associated coronavirus (SARS-CoV), RHDV] and dsRNA virus [Infectious bursal disease virus (IBDV), Infectious pancreatic necrosis virus(IPNV), bacteriophage Փ-6] RdRps, is not present in the highly conserved motif G of tripartite cryptoviruses (FcCV, RmCV, RoCV-1, and RsCV-2) including ArCV-1. Proline is substituted by glycine (Gly123), suggesting some flexibility at this position. It has been described that the residues of motif G contact the nucleic acid at its 5’ template overhang and form part of the channel for the template strand observed in the structures of reovirus and bacteriophage Փ-6 RdRps [12, 13], and the motif also predicted to be involved in positioning of the 5’template strand [21].

**Fig 5:**
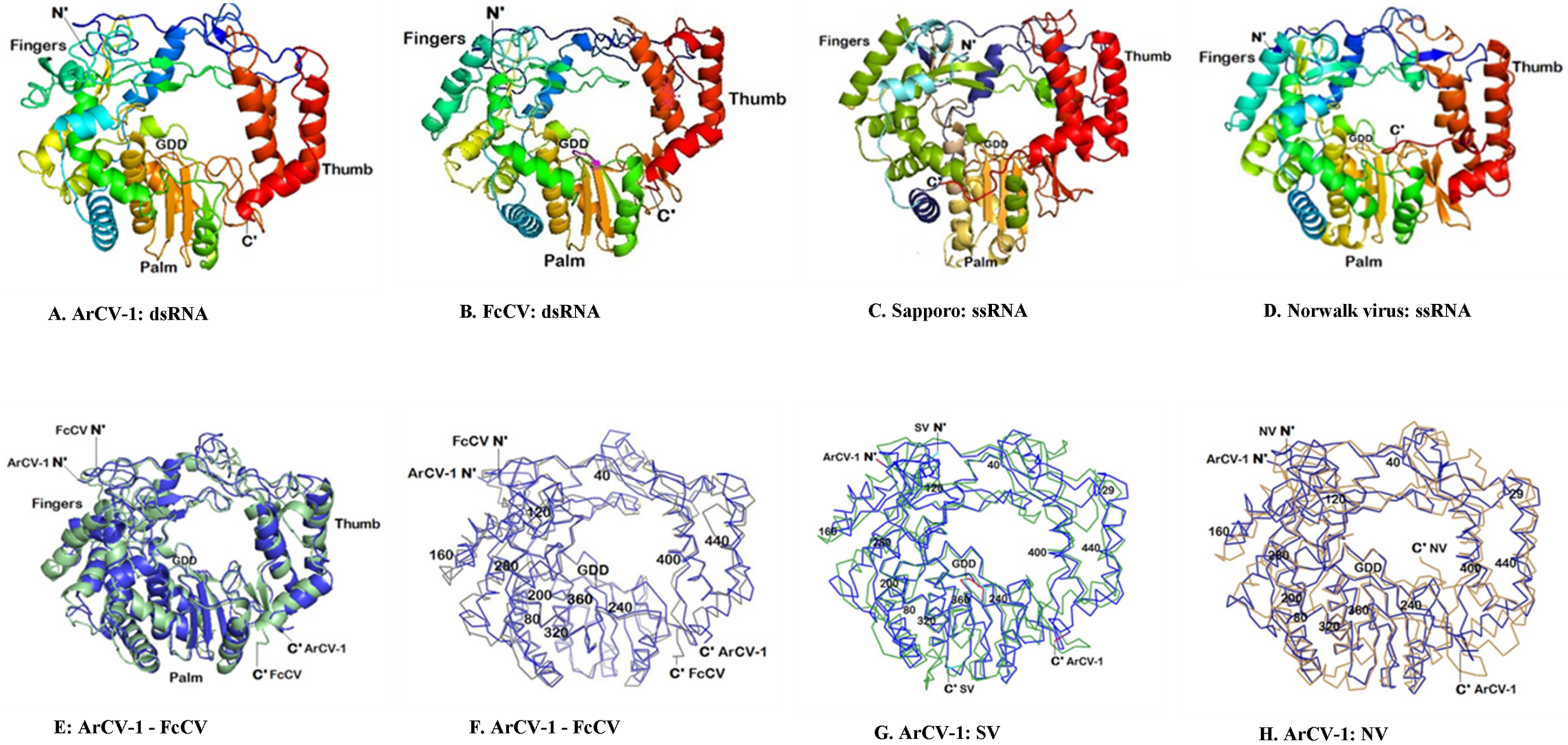
Comparison of cryptoviral and picornaviral polymerase structures. **(A)** Cartoon representation showing ArCV-1 3Dpol; **(B)** *Fragaria chiloensis cryptic virus* (FcCV) **(C)** Sapporo virus (SV; PDB ID, 2wk4A) and **(D)** Norwalk virus (NV; PDB ID 1SH0). The molecules are shown in conventional right hand orientation with spectrum colors. The N-terminus colored blue and red for C-terminus for all the molecules. The three subdomains the fingers (shades blue and green) palm and thumb domains are in yellow and red. Carboxyl-terminal ends of the ArCV-1, FcCV, and SV were seen protruded out and interestingly in NV C-terminus was found to be located in the catalytic region of the central cavity. **(E)** ArCV-1, 3Dpol (blue) in a cartoon representation was superimposed by FcCV (green) polymerase. **(F)** in a backbone representation ArCV-1 3Dpol (blue), with every 40th residues numbered was superimposed by FcCV (grey) polymerase and **(G)** by Sapporo virus (green) polymerase (PDB ID, 2wk4A) and **(H)** by Norwalk virus (brown) polymerase (PDB ID, 2b43D) A high degree of alignment was noticed ArCV-1, 3Dpol with FcCV and SV followed by NV especially in the palm region. Structural comparison analysis provided for SV and NV an RMSD value of 2.10 Å and 2.15 Å, respectively.

The palm subdomain(residues 233–251 and 310–398) consists of three β-sheets (β-3, β-6, β-7) sandwiched between helices α-11 and α-12 and a long loop from α-12 connecting α-13 in the thumb, α-7 being at the junction of palm and thumb (Fig 4A). The palm domain shows the maximum conservation in the conserved motifs A, B, C, D and E (S1 Fig, Fig 4B), and has been shown to play different roles in the catalytic activity of the enzyme, found in most of the known RdRps [20, 28]. Palm sub-domain contained the structural architecture to suit to the functions, has remarkable alignment with the known RdRps. ArCV-1 RdRp sequence identity with cryptoviruses is high, like the structural alignment. Interestingly, with less sequence homology (Table 4) a close structural alignment was noticed between ArCV-1 and Sapovirus (SV) and Norwalk virus (NV) (+ssRNA) RdRps as evident from the panels shown in Fig 5E–5H. Residues playing key roles in active site interactions, distributed across the motifs A to E, in different classes of RdRps have been identified in ArCV-1 dsRNA RdRp as well as the RdRps of other cryptoviruses have been discussed as below.

**Table 4:** Structural sequence identity ranking of ArCV-1 RdRp to the top five identified structural analogs in PDB.

Rank	RdRp	PDB ID	TM Score	RMSD	%residues*
1	Sapovirus (SV)	2WK4	0.897	2.10	15.1
2	Norwalk virus (NV)	2B43D	0.895	2.15	15.5
3	Rabbit hemorrhagic disease virus (RHDV)	1KHW	0.893	2.12	13.0
4	Murine Norovirus (MNV)	3NAI	0.886	2.23	12.5
5	Foot-and-mouth diseasevirus (FMDV)	1WNE	0.837	2.62	13.2

Rank = Ranking of proteins on TM-score of the structural alignment between ArCV-1 RdRp structure and known structures in the PDB library.

TM Score = Template modeling score, is a measure of sequence identity between two protein structures with different tertiary structures.

RMSD = Root mean square deviation represents the differences in sample standard deviation between predicted values and observed values.

* = % sequence identity of amino acid residues (by Clustal W 2.0)

Motif A consists of conserved residues present between the β-sheet 3 running into α-helix 8. The RdRp contained active residues D242, F246 and D247 are conserved in motif A of several picornaviruses. Motifs A and C located in the 3D^pol^ structurally adjacent to each other contain aspartate residues (D341), involved in a two-metal (Mg2^+^ and/or Mn2^+^) mechanism of catalysis [48–50]. Motifs A, B, and F are key for nucleotide triphosphate (NTP) binding, motifs A and C are also involved in binding of metal ions [51].

**Fig 6:**
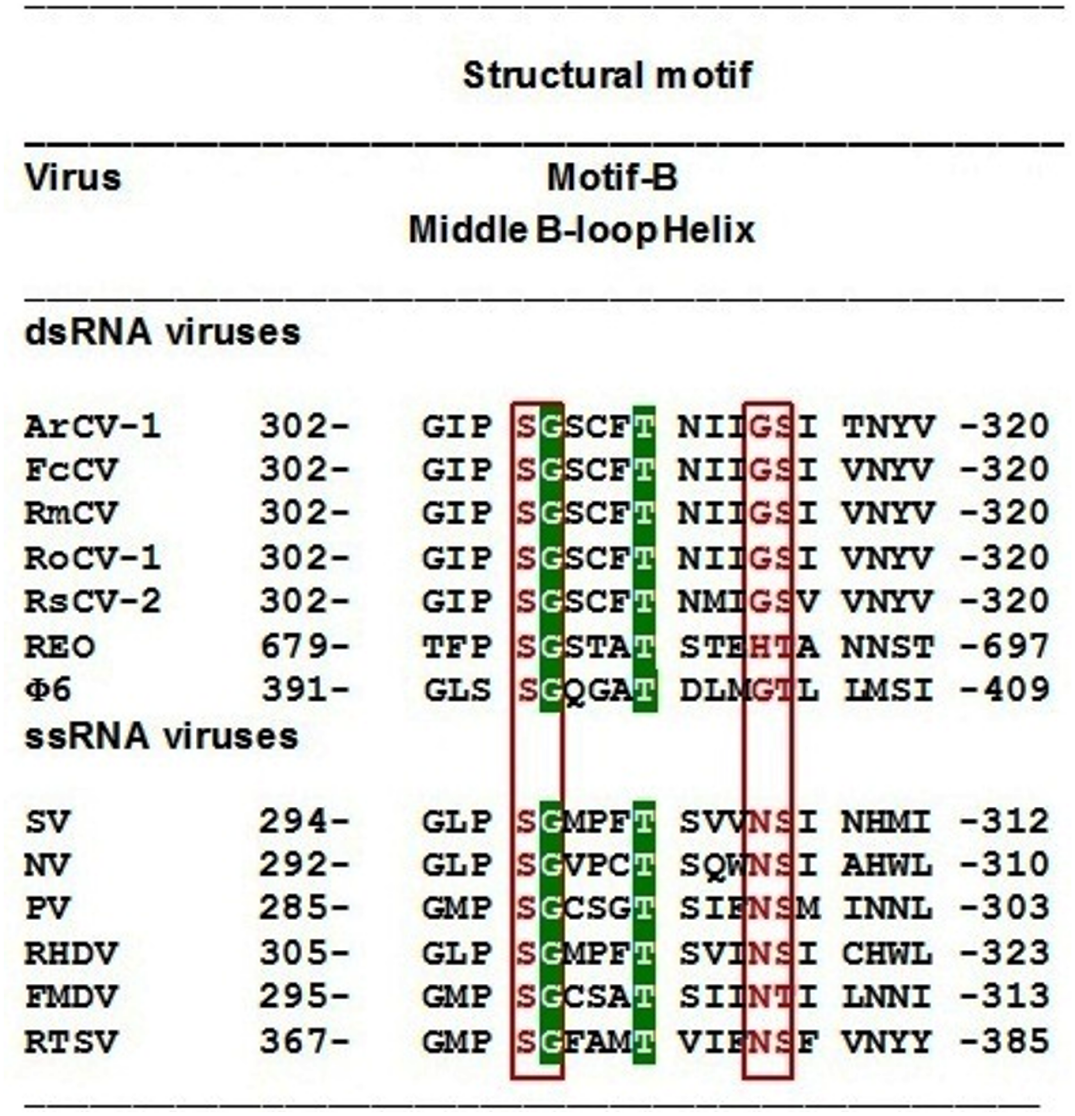
Conserved residues the RdRp B motif of ds RNA cryptovirus and ss (+) RNA picornaviruses. The RdRp multifunctional B motif, comprising of conserved residues, forms structurally important entity containing B-loop (see Fig 5F) as demonstrated in picornaviruses. Shown here is a similar motif in cryptovirus RdRps with the conserved gly306 residue that acts as axis for the conformational changes, highlighted in green block. The positions of the catalytic Asn (in α-helix11, predominantly substituted by gly314 in dsRNA viruses) and of the Ser and Thr residues of picornaviruses interacting with the incoming NTP substrate also are common to cryptovirus RdRps (details in Text). ArCV-1 (HG797710), FcCV (DQ093961), RmCV (EU024675), RoCV-1 (EU413666), RsCV-2 (DQ218036), Phi6 (PDB 4A8W), Reovirus (RV; PDB 1MUK), SV (PDB 2WK4), NV (PDB 2B43), RHDV (PDB 1KHW), FMDV (PDB 1WNE). Middle = middle finger of the fingers subdomain; Loop = structures in palm subdomain.

**Motif B:** It consists of conserved residues as a single block (S1 Fig) containing Ser305, Cys308, Thr310, Ile312 and Asn311 (α-helix 11; Fig 4) and are predicted to play a major role in the catalytic process conserved in picornaviruses as well (Fig 6). In the N-terminal of the motif, the coil forms a loop commonly referred to as B-loop (Ser, Gly, Ser and Cys), connecting to the long α-helix11. Motif B provides important orienting interactions with the incoming deoxyribonucleoside rNTP [52], and the B-loop has been described structurally flexible and moves its conformations [53], the key residue glycine (G306 in ArCV-1) was described to act like a “hinge” for the movement. Substitutions of this glycine residue in 3Dpol basically abolished RNA synthesis *in vitro* [35]. The high sequence and structural conservation of the B-loop among viral RdRps have been reported [17]. Rearrangement residues (SGSCFTNIIGSI) of ArCV-1 motif B which are conserved in cryptoviruses (S1 Fig and Fig 6) did not affect the structural sequence identity of the motif with picornaviruses. While describing the function of B-loop in FMDV the authors speculated that the rearrangements in the template channel and the B-loop occur in a concerted manner and that these changes collectively serve to regulate RNA replication, processivity and fidelity, and the active residues in motif B, each has a definite role to play [17].

Motif B takes part in template binding, incoming nucleotide recognition [39] and determining whether RNA or DNA will be synthesized by selecting rNTPs and dNTPs and correct positioning of the sugar in the ribose-binding pocket [54], asparagine residue thought to be playing a vital role in its functioning. Briefly, Asn residue in this motif (Asn307 in FMDV) has been confirmed to interact with incoming rNTP (play a role in differentiating between dNTPs and rNTPs and thus determines whether RNA or DNA is synthesized) during RNA elongation in a number of picornavirus elongation complexes [39, 49, 54–56]. Similar positioning of Asn residue (N297) is present in the long helix α-H [39], 6.6Å away from the active aspartate of the catalytic center. However, the highly conserved Asn residue which is critical for enzymatic activity was not evident in motif B of the ArCV-1 RdRp (Fig 6). This is a conspicuous difference between the RdRps of picornaviruses and cryptoviruses. The Asn residue appears to be substituted by glycine (G314), present at the same position in the α-11 at a distance of 6.9Å away from the active aspartate of the catalytic center and is conserved in most of the dsRNA cryptovirus RdRps and also exist in phage Փ-6 motif B (Gly403). Crotty et al. [49] demonstrated that an absolutely conserved residue (Asn) of motif B within the nucleotide binding pocket of the poliovirus polymerase can be substituted for a different amino acid, yielding replication-competent virus. Glycine that replaced the Asn in cryptoviruses does not have amide group [49]. Employing one of the PV mutants (PV-297G) developed; authors explained that the molecular modeling suggests that a glycine at position 297 leaves sufficiently a large pocket for an additional water molecule [49]. Therefore, glycine may substitute for asparagine 297 by allowing a water molecule to become the hydrogen bonding partner for the NTP 2′OH. These results attest to the extreme evolutionary flexibility of the viral RdRp, in terms of both structure and cation usage [49], and could explain, devoid of Asn (Fig 6), in motif B of ArCV-1 and other cryptoviruses the polymerase is able to function.

Motif C is comprised of β-sheets-6 and 7 in an anti-parallel manner connected by a loop towards the central cavity and this loop bares the highly conserved Gly340-Asp341-Asp342 observed in most of the RdRps, now observed in plant cryptic virus RdRps. Like the B-loop the beta bend GDD motif is structurally similar in all classes of RdRps, described as the center of catalytic activity of the enzyme in coordinating the metal ion. The first aspartate (Asp341in ArCV-1) is known to facilitate the rNTP transfer and by binding to the metal ion at the catalytic site. It has been shown that first N-terminus aspartate is absolutely required for enzyme function [29, 32, 35, 57, 58]. The presence of the second aspartate almost is universal in GDD motif with a couple of exceptions [replaced by asparagine (ADN) in IBDV and glutamate (GDE) in case of KF of *E*. *coli* DNA polymerase I] signifying flexibility at this position. But any substitutions made in the second aspartate were not tolerated as reported in poliovirus RdRp [35, 58]. Phage Փ–6 and IBDV (dsRNA) RdRps contain the catalytic motif as SDD and ADN respectively that makes the requirement for the glycine residue of GDD motif also somewhat flexible *in vitro*. However, substitutions at glycine position in Encephalomyocarditis virus(EMCV) 3Dpol make the RdRp completely inactive *in vitro* [35], as in the case of potatovirus X (PVX) RdRp *in vivo* were not tolerated [57], suggesting that the requirements for this position may be more strict [59], with some RdRps to function. Aspartate in Motif A interacts in catalytic reaction as they, together with the first aspartate of GDD, coordinate the rNTP transfer [60]. ArCV-1 3Dpol contains a couple of highly conserved aspartate “active residues” in motif A; D242_A_ and D247_A_ present at a distance of 12.4Å to each other (S3 Fig) and the D242_A,_ 7.0Å from D341 are similar to those referred above [60], could be interacting with Asp341 by coordinating catalytically essential metals as noticed in many RdRps [23, 39, 61]. Many of the active residues of the conserved motifs (S1 and S2 Figs) present in the central conserved region of the RdRp were located in the central cavity or close by. This region forms a domain that is partially resistant to protease digestions [59].

Motif D has been identified in ArCV-1, 3Dpol in a long loop between α-helix12 and α-helix13 (Fig 4A and 4B). A positively charged residue lysine373 along with serene is conserved in this motif (D**KS**D) across all cryptoviruses (S1 Fig). Lysine seems to be sustained in the sequences of motif D of all RdRps [20]. Motif D is inconsistent and not so well conserved in members of the family *Picornaviridae* and the cryptoviruses analyzed in the present study. Structural variability was noticed in the reported RdRps as well, but the majority showed motif D in a coil. The N-terminal Asp372 in the motif of the 3Dpol was conserved in many primer-dependent RdRps of calciviruses and picornaviruses, but not in the rest of cryptoviruses and in *de novo* RdRps (dsRNA-phage Փ-6, IBDV and reovirus). Active residues in motif D have been postulated to play critical roles in catalysis [62]. Motif D for long considered “scaffolding” for the palm, keeping the structural integrity [39, 59]. Movement of motif D facilitates active site closure and is sensitive to incorrect NTP binding and mismatches at the RNA terminus. These studies link the conformation of motif D to the efficiency and fidelity of nucleotide addition in elongation [63]. Closing and reopening of the active site happens as a result of association of motif D with motif A, which undergo coordinated conformational changes [64].

Motif E was located at the junction of the palm and thumb, and was described as not integral to the conserved core structure [39], contains characteristic motif XFL where leucine was predicted as active residue. The conserved sequence at amino acids position 384 to 388 (TFLSR) was detected in the ArCV-1 3Dpol at the mentioned location α-helix turn α-helix as part of the long moving loop structure, adjacent to motif D close to the catalytic center (Fig 4B and 4C). There was variation in the amino acid T384 position in the cryptovirus primary sequence (S1 Fig) as well as in picornaviruses. The hydrophobic L386 residue of this motif was found in the analysis to be active residue; described essentially for catalytic activity. This motif described interacts with the elements of β-sheets of the palm core structure and the elements in the thumb subdomain in both poliovirus RdRp and RT [39, 65]. Substitution in the vicinity of active leucine residue is not tolerated in polio virus and an insertion of a leucine after R376 of PV (R388 in ArCv-1) was shown to abolish replication *in vivo* and *in vitro* [30], and a double mutant K375A/R376A of the same virus was found to abolish viral replication *in vivo* [66], suggesting that the critical role these amino acids play in RdRp functioning.

Thumb subdomain (residue 399 to residue 475) constitutes mainly of helical structures. Out of four α-helices, α-13 and α-14 helices adjacent to each other runs antiparallel while α-16 helix runs across and ends with protruding Gln 475 residue (Fig 4A). The α-13—α-14, and α-15—α-16 are interconnected resulting in larger loops at the crest of the thumb subdomain and bending over forward to tie into the N-terminal region of the finger tips, creating a complete annulus around the catalytic site on the palm (Fig 4A). The small size of the domain contributes to the formation of a large central cleft [45], which is located at the front of the molecule (Fig 4A, 4C and 4D) and leads to the active site shown in the surface representation of the molecule. ArCV-1, 3D^pol^ visually seems to be having similar central cavity architecture and dimensions with FMDV, PV and HRV-16. The C-terminus region is conserved amongst the cryptoviruses studied showing conserved residues in motifs that are not observed in the single stranded picornaviruses or in the dsRNA viruses. CLRML (402–06) motif as part of α-13, facing the central cavity, the rest YPEY (408–11), DAG (429–31), and WPD (461–63) motifs are present in the remaining 3 helices.

The overall topology of ArCV-1 3D^pol^ smaller thumb subdomain structure resembles more with ssRNA (+) RdRp analogs of NV, SV, HRV, RHDV, PV and FMDV than the *de novo* dsRNA viruses. The main structural differences between RdRps of ArCV-1 and the closely related FcCV and RoCV-1, RmCV, RsCV-2 (*Deltapartitivirus*) are in the thumb region (Fig 5A–5D). The thumb subdomain of these cryptoviruses contained four α-helices (α-α-α-α) on the contrary FcCV thumb had an extra helix making total thumb α-helices to five (β-β-α-α-α-α-α). The second difference noticed was the palm and thumb interface region. ArCV-1 3D pol, consists of long coiled loops connecting α-12 (palm) and α-13 (thumb) with no β-sheets in this region. While the RdRps of other cryptoviruses consists of two antiparallel β-sheets followed by motif E as was observed in NV and SV (Fig 5A–5D) making the thumb configuration as β-β-α-α-α-α.

The thumb C-terminus observed in ArCV-1 3Dpol was protruded out away from central cavity like other cryptoviruses except in case of RsCV-1 (*Alphapartitivirus*) where the C-terminus helix (α-16) extends in to a compact tuft of coil packs against the front face of the molecule and the end pointing towards the central cavity, a similar structure (S3 Fig) seen in rice tungro virus [RTSV; ssRNA (+)] a plant picornavirus and NV (S3D Fig, Fig 5D). The C-terminus of RmCV (EU024675-77) genomic segments has a unique poly “A” tract. Flaviviruses, IBDV, Bacteriophage Փ-6and Reo (dsRNA) viruses with larger polymerase molecule contain elaborate thumb architecture with 7 to 15 α-helices and 2–4β-sheets. The phage Phi6 pol C-terminal extends back ploughing parts of it through the molecule and ends in the middle finger region of the other side [12]. Interestingly, substitutions or deletion of specific residues in the thumb subdomain of Brome mosaic virus (BMV 2a) and poliovirus 3Dpol abolish RNA replication [30, 67].

### Structural comparison with other RdRps

The analysis showed ArCV-1 RdRp structural alignment with the viruses belonging to *Picornaviridae* and *Deltapartitiviridae* (Fig 5 and S3 Fig). The range of identity varied with the ranking viruses which had high identity with the RdRp of ArCV-1. Different genera of picornaviruses showed high template modeling (TM) score with ArCV-1 3D pol; NV (0.898) [68], SV (0.898) [69],Rabbit hemorrhagic disease virus (RHDV) (0.895) [70], all members of the family *Caliciviridae* (Fig 5G and 5H) and with moderate TM score with alignments of viruses members of the family *Picornaviridae*; FMDV (0.834) [33], EMCV (0.830) [71] and Poliovirus (PV) (0.823) [39]. These economically important viruses are human and animal pathogens and the structural details are available in PDB archives which doesn’t have the information on the RdRps of cryptic viruses to compare. For the first time using computer analysis we are reporting structural details of RdRps of few plant cryptic viruses with high structural similarity to ArCV-1 3Dpol (Fig 5E and 5F). Plant cryptovirus and human picornovirus RdRps that showed structural identity were superimposed on ArCV-1 3D RdRp in a cartoon and backbone representations. ArCV-1, 3Dpol (blue) was superimposed by FcCV (green) RdRp (Fig 5E) in a cartoon representation showed exact structural alignment. Similar high-level alignment was noticed when ArCV-1 3Dpol (blue), superimposed by FcCV (grey) RdRp (Fig 5F), by SV (green) RdRp (PDB ID, 2wk4A) (Fig 5G) and by NV (brown) RdRp (PDB ID, 2b43D) (Fig 5H) in a backbone representation. Every 40^th^ residue from N-terminal to C-terminal was numbered in a stereo back the bone representation of the RdRps. A high degree of alignment was noticed ArCV-1, 3Dpol with FcCV and SV followed by NV especially in the palm region containing the A to E motifs with conserved residues. Structural comparison analysis provided for SV and NV an RMSD value of 2.10Å and 2.15Å, respectively. Our findings validate the analysis predictions and reveal a possible evolutionary linkage between cryptoviruses (dsRNA viruses) and the picornaviruses (+ssRNA viruses).

#### Phylogenetic analysis

**Fig 7:**
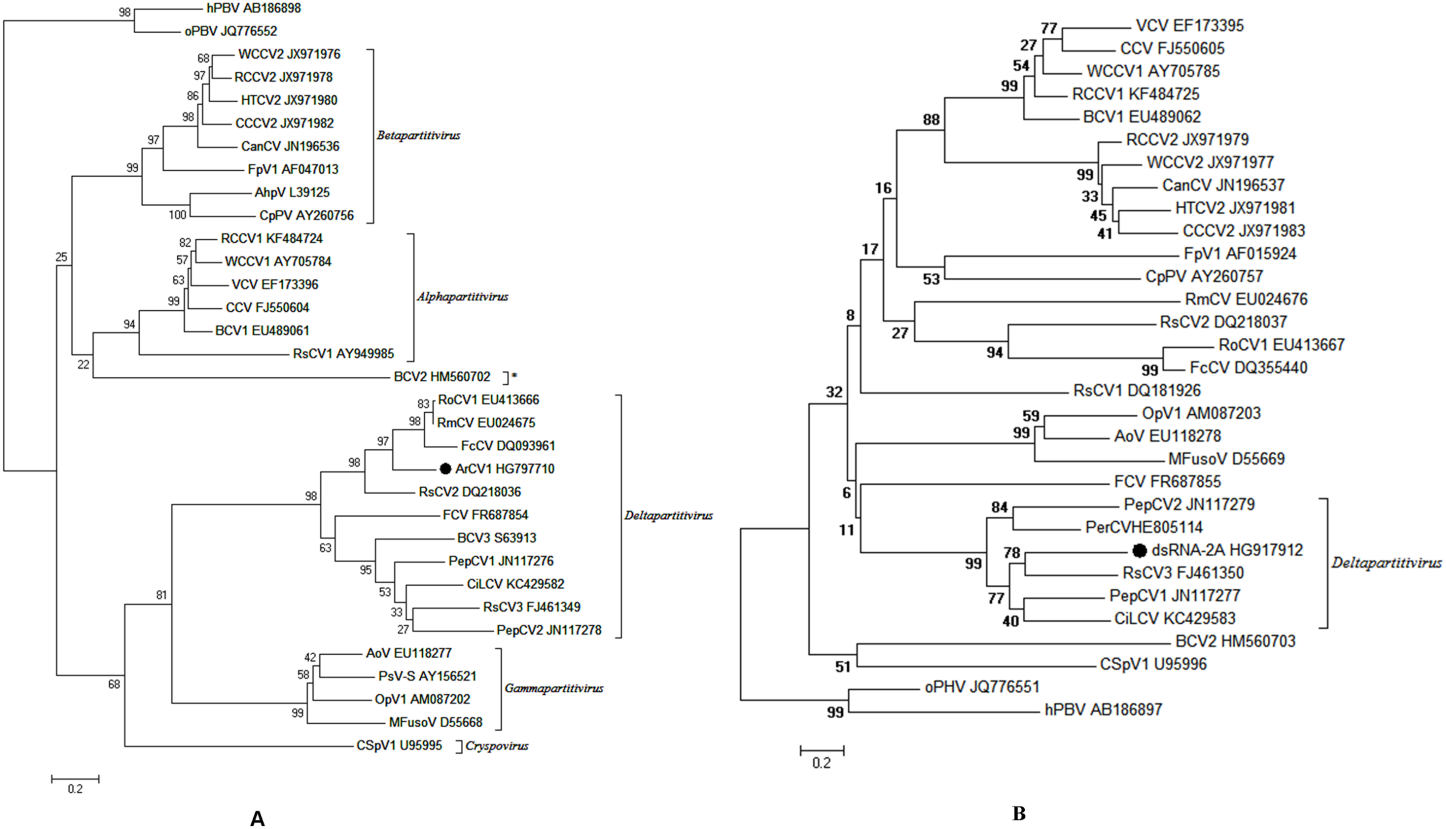
Phylogenetic analysis of ArCV-1 dsRNA1 (RdRp) and dsRNA-2A. **(A)** Phylogenetically though all members originate from common ancestor as they diverge from a single clade formed five distinct groups corresponding to their genera (Group 1- *Alphapartitivirus*; Group 2- *Betapartitivirus*; Group 3- *Gammapartitivirus*; Group 4- *Deltapartitivirus* and Group 5- *Cryspovirus*). Members of group 1 and 2 clustered separately but originate from common point however origin of members of group 3 were different. Group 4 and 5 originate from common sub-clade, which is different from other groups. ArCV-1 RNA-1 clusters with tripartite members of the genus *Deltapartitivirus* and is closely related to FcCV and RsCV-2. *Beet cryptic virus-2 was an assigned member of *Deltapartitivirus* but it was noticed to fit in with *Alphapartitivirus*. **(B)** Phylogenetic analysis of dsRNA-2A (P4 protein), displays its proximity with bipartite PepCV-2 and RsCV-3. The bar represents base substitution per site. Sequences of different partitiviruses from NCBI database were used in the analysis (S1 Table). RdRps of hPBV (AB186898) and oPBV (JQ776552) and CP sequence (hPBV: AB186897 and Q776551) were used to provide an out group root in the analysis.

BLAST analysis involving several tripartite cryptoviruses revealed that the ArCV-1 genome shared a low level of identity with the orthologs, mentioned in Table 2. Besides 5’ terminus 16 nt similarity, a high degree of sequence homology between the ArCV-1 5’NTR sequences of the three genome segments was noticed while lacking decipherable identity in the 3’proximal un-translated regions. The genomic segments of several *Partitiviridae* members usually have sequence homology in the terminus ends [72, 73]. ArCV-1 RdRp (dsRNA-1) and dsRNA-2A sequences were used in the phylogenetic analysis with corresponding sequences of members of the genera *Alphapartitivirus*, *Betapartitivirus*, *Gammapartitivirus*, *Deltapatitivirus* and *Cryspovirus* (Fig 7) mentioned in the S1 Table. The analysis revealed that the viruses used in the study assembled in five groups, as recognized previously [8]. ArCV-1, dsRNA-1 clustered with tripartite RoCV-1, FCV and FcCV and with members of the genus *Deltapartitivirus* (Fig 7A). Phylogeny of dsRNA-2A revealed close relationship with members of bipartite *Deltapartitivirus*; PepCV-1, PepCV-2, RsCV-3 and CiLCV (Fig 7B). The findings further support the hypothesis that dsRNA-2A does not belong to the tripartite ArCV-1.

## Discussion

In this study, the complete sequence of four dsRNAs isolated from asymptomatic pigeonpea plants was determined. Three dsRNA segments formed the genome of a new cryptovirus and named Arhar cryptic virus-1 (ArCV-1). The fourth dsRNA (dsRNA-2A; P4) was not related to the ArCV-1 genome and has characteristics like a viral coat protein. ArCV-1 dsRNA-1 dsRNA-2 and dsRNA-3 have been identified as encoding RdRp, CP-1 and CP-2, respectively (Fig 2A). Concentration of dsRNAs varied, dsRNA-1 (RdRp) was the most predominant transcript followed by the two capsid proteins (Fig 1). The ArCV-1 genome segments shared high degree of sequence identity in the 5’NTR including the marker 5’terminus 16 nt conserved sequences. Similar conservation in the tripartite viruses (Fig 3) was noticed; FcCV, RoCV-1, RsCV-2 [7, 74, 75], and were clustered together phylogenetically to form the genus *Deltapartitivirus*, a member of the family *Partitiviridae* (Fig 7A). The 3' NTR (50 nt) was rich in GC content and the three dsRNAs of ArCV-1 terminated with TC (Fig 2, S1 Fig). Furthermore, these findings were supported by a phylogenetic analysis which revealed that ArCV-1 formed a unique phylogenetic cluster with FcCV, RmCV and RoCV-1 within the genus *Deltapartitivirus*, infecting different genera of the family *Rosaceae*.

The status and role of dsRNA-2A which encodes 44.5kDa protein (P4), which was found in least concentration, is not known. Association of an additional dsRNA segment (s), discrepancies with cryptic virus infections was not uncommon. In an earlier finding, the association of an extra component in many *Vicia faba* cultivars infected with Vicia crypticvirus (VCV) was reported [72, 76]. While characterizing the cryptovirus infection of radish (*Raphanus sativus-root cv*.Yidianghong) the authors suggested that more than one partitivirus was co-infecting radish leaves [73, 75]. The second virus in the complex has two smaller segments (RasR4 and RasR5) contained conserved 5’ termini sequence with RasR3 (RdRp) deem to be with unknown functions not having sequence identity with the known capsid proteins. In the present study, the four dsRNA shared sequence identity with members of *Deltapartitivirus*. The dsRNA-2A, (P4) contains sequence that resembles viral coat protein showed an unusually long 3'-terminal 353nt NTR region. The 5’-terminus of this dsRNA is different, does not share sequence identity with the 5’-terminus conserved genome segments of ArCV-1. However, P4 showed sequence identity with bipartite and tripartite cryptovirus coat proteins with pronounced resemblance to the bipartite cryptoviruses (Table 3). Phylogenetic analysis indicated that the P4 was closely related to RsCV-3 segment-2; coat protein (FJ461350). This indicates that the origin of P4 is related to *Partitiviridae*, but we have no evidence that P4 may interact with ArCV-1 in its replicative cycle or it is a derivative of rearranged sequences of the ArCV-1 coat proteins-highly unlikely or it could be part of the genome of evolving yet another cryptic virus.

Primary sequence analysis of ArCV-1 RdRp revealed, besides several conserved sequences amongst the cryptoviruses; at least seven conserved signature motifs in a particular order which were well characterized [20, 28, 59]. These motifs were aligned with the RdRps of picornaviruses ssRNA (+) that led us to examine the structural similarity, if any, of ArCv-1 3Dpol with known RdRps in further detail. However, detailed studies of RdRps encoded by partitiviruses have not been reported. Amongst the double-stranded viruses studied; representatives of *Cystoviridae* [12], *Reoviridae* [13], and *Birnaviridae* [14, 77], in terms of the structure, mechanism of initiation of replication and transcription by their active RdRp was explained with the 3D structural analysis.

This report illustrates several interesting features of the ArCV-1 RdRp and a cryptovirus 3D^pol^ and full-length structure was determined for the first time. It was revealed that the 3D^pol^ shared structural details more with calciviruses, members of the family *Picornaviridae*. Computational analysis was used to develop and characterize ArCV-1 3D ^pol^ structure at 7.9 Å resolution. We studied several RdRp analogs in the PDB which showed homologous structural identity with ArCV-1 RdRp mostly of animal and human picornaviruses. Unlike the genomic RNA of picornaviruses, the ArCV-1 dsRNA-1 that encodes RdRp (55kDa) protein does not show 5’ cap or VPg or 3’ polyadenylation, but a stretch of NTRs present on both the termini with no internal structures. The 3Dpol structures of Norwalk virus, Poliovirus and Sapporo virus which were previously determined and the respective sequences used, produced identical 3D structures in our computer analysis, confirming authenticity of ArCV-1 structural analysis.

The overall topology of the ArCV-1 RdRp structure resembles the general description of the three-dimensional structures of the primer dependent initiating single stranded RNA (+) RdRps [33, 39, 45, 68–71, 78–80], depicted as a closed right hand (Fig 4A), differentiated as fingers, palm and thumb sub domain [52]. 3D^pol^ structure of ArCV-1 and other cryptoviruses analyzed are found to be different from the dsRNA viruses; bacteriophage Փ-6, Reovirus and IBDV [12–14, 77]. In general, the topology of the cryptoviruses; BCV-2, FcCV, RmCV, RoCV-1, RsCV-2, RsCV-3 (*Deltapartitivirus*) and RsCV-1 (*Alphapartitivirus*) RdRps, had close identity with ArCV-1 with minor differences. The ArCV-1 RdRp sharing 72.4% sequence identity with FcCV and only 14.8% sequence identity with RsCV-1 (Fig 4). Despite low amino acid identity (15%), interestingly the ArCV-1 RdRp shares more structural identity with the members of the family *Caliciviridae*; NV and SV, (Fig 5, S3 Fig). The structural identity of NV at 2.17 Å resolution (1SH0, 2B43) and SV at 2.3 Å resolution (2CKW) 3D^pol^ with the ArCV-1 RdRp, indicate a closer evolutionary link between the very divergent groups of viruses, and the polymerase has no structural alignment with other dsRNA, bacteriophage Փ-6, reovirus and IBDV which have larger RdRp molecules with complex dense subdomain structures.

The analysis of RdRp sequence and structure revealed the occurrence of motifs A to G which play an important role in NTP binding and catalysis [70], described for *Calicivirus* and *Picornavirus* RdRps has structural similarity (Fig 4B) as described earlier. The similarity was confirmed by the visual observation of their superimposed structures (Fig 5E and 5F). The unique feature noticed in ArCV-1 RdRp is an N-terminal portion of the fingers reaches to the thumb bridging the fingers and thumb sub domains [43], was observed with all cryptoviruses studied. This feature was noticed in ssRNA (+) and dsRNA RdRps visualized in less cluttered IBDV RdRp. Fingers subdomain contains motifs F and G. Motif F of cryptoviruses unlike the other classes of RdRps is well conserved and extends from few residues before β-sheet 2 to α-helix 6 and the connecting coils. Computer analysis predicted that Motif F contains several active residues supposed to be interacting with the RNA in the ArCV-1 3D complex were detected (Fig 5B, green colour, S1 Fig). Basic residues Lys181 and Arg183 of this motif were also conserved in other RdRps; for example, in FMDV motif F, Lysine residue along with two arginine residues (K172, R168 and R179) predicted to interact with the incoming NTP substrate [33]. Motif G forms part of a long coiled loop connecting α-helices 4 and 5 in the fingers subdomain (Fig 4B, red). Motif G is highly conserved in the cryptoviral RdRps. The conserved Gly121 and Lys128 (in ArCV-1) of this motif in all classes of RdRps seem to be a strict requirement of motif G.

Mutational analyses in PV and FMDV also have demonstrated that some substitutions in residues located far from the active site, in particular at the RdRp N-terminus, have significant effects on catalysis and fidelity [17]. Several highly conserved stretches of sequence motifs of ArCV-1 in the N-terminus to the central region, have been identified uniquely to cryptic virus RdRps. Motifs 1 and 2 are observed in the RdRp sequence away from the palm subdomain could play a role in catalysis (S1 Fig).

The palm sub domain contains A to E motifs in a series. β-sheet 3 to α-8 consists of residues 241–251 were identified as Motif A (Fig 4B, blue color) contains conserved Asp242 and Asp247. Like SV and NV, RHDV 3D^pol^ also has a high degree of structural homology with ArCV-1 (Fig 5 and S3 Fig) and the aspartate residues share identical positions in motif A and C (Asp_A_250 and Asp_C_354 in RHDV). It has been described that metal ions that are likely to be involved in the nucleotide transfer reaction, interact with aspartate residues at positions 250, 354, and 355 in RHDV [70]. Similarly, in ArCV-1 3D^pol^ the corresponding two aspartate residues Asp_A_242 and Asp_A_247 which are separated by a distance of 12.4Å and Asp_A_242 being closer (7.0 Å) to the catalytic aspartate Asp_C_341, could be performing the same functions. Ser, Gly, Thr and NS/T are identified as highly conserved active residues in motif B (Fig 4B, purple), each with specific function in many picornaviruses. Asn residue (Asn307 in FMDV) of motif B plays a role in differentiating between dNTPs and rNTPs and thus determines whether RNA or DNA is synthesized [39, 55]. Later it was determined that the active Asp residue of motif A, and Asn of motif B (Asp240, Asn307 in FMDV) play a critical role in rNTP selection as evident from the FMDV replication process [33]. Asparagine residue is highly conserved in the long helix, about 6.6Å away from the active aspartate of the catalytic center in picornaviruses. On the contrary, the Asn residue was substituted by Glycine (314) present almost at the same position (Fig 6), in the α-helix (α-11 in ArCV-1), conserved in most of the dsRNA cryptovirus RdRps. In poliovirus mutational studies, Asn (297) was substituted by glycine (PV-297G) resulted in replication-competent virus, which probably explains how cryptoviruses are able to multiply with altered functional residue (Gly314) involved in rNTP selection (S3A Fig) [49]. Rearrangement (SGSCFTNIIGSI) of few residues of the ArCV-1 motif B which are conserved in cryptoviruses did not affect the structural similarity of the motif with picornaviruses. The architecture of the palm subdomain of ArCV-1 3D^pol^ with three β-Sheets (β-3, β-6 and β-7) sandwiched between two α-helices (α-7 and α-12) is highly conserved feature in most of the RdRps as motif C (Fig 4B, pale pink). The anti-parallel β-Sheets 6 and 7 connected by a short loop containing GDD (Asp 341 and Asp 342) sequence described as catalytic center. Structural properties and catalytical aspects of palm and thumb (residue 399 to residue 475) sub-domains are discussed in greater detail in the results section.

Understanding structural details of known viral RdRps and associated mechanism described for the initiation of RNA synthesis and chain elongation reveal, that these viruses use different strategies to initiate replication. It has been described that viral replication can be initiated by two principally differing mechanisms; the primer-dependent [42], and primer independent or *de novo* initiations [81–83]. However, Influenza virus employs a combination of the two mechanisms with the choice being determined by the type of RNA to be synthesized [84]. The *de novo* synthesis involving the interactions of required components, the initiation essentially a one-nucleotide primer provides the 3’-hydroxyl for the addition of the next nucleotide [42]. The *de novo* RdRps have specific structural elaborations that function to stabilize the initiation complex. Such initiation platforms have been found in the crystal structures of viral RdRps of *Cystoviridae*, *Reoviridae* and *Birnaviridae* [12, 13, 77]. In the second mechanism, the primer-dependent initiation requires the use of either an oligonucleotide or a protein primer to provide the hydroxyl nucleophile. Members of the *Picornaviridae* family use exclusively the protein-primed mechanism of initiation [17, 81].

Viruses belonging to different groups containing ssRNA (+), dsRNA as genomes adopt *de novo* initiation mechanism. For example, members of the genus *Flavivirus*: hepatitis C virus (HCV) [85], and dengue virus (DENV-2) [86], as well as Enterobacteria phage Q beta (Qb) [87], all have ssRNA (+) genome. The second group to adopt *de novo* initiation are the dsRNA viruses namely, cystoviral bacteriophage Փ-6 [12], Infectious bursal disease virus(IBDV) (family *Birnavirdae*), [14, 77], and reovirus (family *Reoviridae*) [13].

By contrast, very little is known about knowledge of the mechanisms underlying dsRNA cryptovirus (*Partitiviridae*) replication as the polymerases of this group have never been studied. ArCV-1 has segmented linear dsRNA genome, each genomic segment is monocistronic: dsRNA-1 that encodes RdRp does not have either 5’ termini or 3’-end structures like template-directed nucleic acid polymerases and does not resemble the vastly intricate 3Dpol structure of known dsRNA bigger molecules that adopt *de novo*, to speculate the polymerase preferred mechanism.

Unlike the *de novo* RdRps, primer dependent RdRps of the viruses of *Caliciviridae* and *Picornaviridae* have significantly smaller thumb subdomains, wider template tunnels and large central cavity with exposed active sites [33, 45] as observed in ArCV-1 3D pol. FMDV RdRp was shown to have the wide enough central cleft to accommodate the bulky priming peptide during the initiation of RNA (complexed with RNA, divalent cation and protein primer VPg), synthesis [46]. Similar structural details of the polymerase mentioned above share a number of unique features by ArCV-1 and other cryptoviruses (Fig 4A, 4C and 4E). If the structural architecture reflects the functions of a polymerase as often supposed [17], the simple thumb organization, arrangement of the motifs (A to G) in a structural order (Fig 4B), large central cavity and the entry and exit channels like in picornaviruses and having remarkable structural conservation with SV, NV and RHDV 3Dpol, lead to a strong possibility that ArCV-1 RdRp could adopt primer dependent initiation mechanism. This important aspect of research of cryptovirus RdRps is yet to be explained. The remarkable structural similarities between ArCV-1 and the other tripartite cryptoviruses (dsRNA) and the positive-stranded RNA viruses of the picornavirus family, in particular, caliciviral RdRps, offer evidence of probable functional and evolutionary relationships between these two virus groups.

Several highly conserved stretches of sequence motifs have been identified that seem unique to cryptic virus RdRp; Motifs 1–2, observed in the RdRp sequence away from the palm subdomain are inimitable and observed in both bi- and tripartite cryptoviruses (S1 Fig), whose exclusive or overlapping functions are yet to be determined. We hope in the near future, biochemical analysis experiments would reveal the function (s) and their relevance in RNA synthesis. Substantial data that has been generated on ArCV-1 3D^pol^ is being authenticated by X-ray crystallography studies and biochemical analysis.

## Material and methods

### Sample collection

Leaf samples of pigeonpea were collected from fields near the Chevella area of Hyderabad; (Telangana state, India). A popular local pigeonpea cultivar *Erra Kandulu*, is being traditionally cultivated in this area which is susceptible to sterility mosaic disease (SMD), fungal blight, and wilt diseases. Leaves from four healthy looking plants, with no disease symptoms, were collected randomly from three fields MG-1, MG-2 and MG-3 (approximately three km apart). Collected leaf samples were designated as Mg-H1, Mg-H2 and Mg-H3 respectively, along with the SMD samples. Leaf samples were placed in separate ziplock bags placed in ice chest with dry ice and transported to the lab. For sample collection, no specific permissions were required from these locations, as these were collected from a private farm land. Also, this study did not involve endangered or protected plant species.

### Extraction and purification of dsRNA

Leaf samples, Mg-H1 Mg-2 and Mg-H3 were used for dsRNA extraction [88]. Briefly, 7g leaf tissue were crushed to fine powder in liquid nitrogen and homogenized in 20 ml of 2× STE extraction buffer (0.1 M Tris-HCl, pH 7.0; 0.2 M NaCl; 2 mM EDTA), containing 1.5% β-mercaptoethanol, 1.5% (w/v) polyvinylpyrrolidone (PVP), 2% (w/v) SDS, and 16 mg of bentonite powder and incubated at 37°C for 15 min. The clarified contents were extracted with equal volume of Tris (pH 8; phenol pH 4.5 ± 0.2) saturated phenol: chloroform: Isoamyl-alcohol (25:24:1) mixture followed by vigorously shaking at room temperature for 20 min. The contents were centrifuged at 11,000 rpm (Heraeus Multifuge 35R+, Thermo Scientific, USA) for 10 min. One fifth volume of ethanol was added to the supernatant and mixed well, followed by the addition of 1g of cellulose CF-11 (Whatman, USA) and the slurry was incubated at room temperature for 30 min with continuous shaking. The mixture was centrifuged at 5,000 rpm for 5 min. and the cellulose pellet with dsRNA was resuspended in 40 ml wash buffer (1× STE with 16% ethanol) and washed twice for 5 min with mild agitation. The cellulose slurry was applied to a 20 ml syringe blocked with glass wool and washed with 20 ml of washing buffer. The bound dsRNA was eluted stepwise (two times) with 15 ml elution buffer (1× STE without ethanol). The eluted dsRNA was further purified by adding 1/5^th^ volume of ethanol and 0.8 g CF-11 cellulose and the suspension was vigorously shaken for 30 min at room temperature. The suspension was transferred to a new 20 ml syringe and the dsRNA was eluted again twice with 2.5 ml elution buffer. The extracted dsRNA was precipitated by adding 1/10^th^ volume of 3 M sodium acetate pH 5.2, and 2.5 volumes of absolute ethanol and incubated overnight at -20°C. The contents were centrifuged at 14,000 rpm for 20 min at 4°C and the resulting dsRNA pellet was washed with 80% (v/v) ethanol, air dried and resuspended in 500 μl MilliQ water. MgCl_2_ was added to the extracted dsRNA to a final concentration of 300 mM and was digested with 500 ng RNase A and 2U DNase I (RNase-free) at 37°C for 45 min followed by phenol/chloroform extraction and precipitating with ethanol. The precipitates were suspended in 500 μl of MilliQ water. Purified dsRNA were separated by electrophoresis on a 1.5% agarose gel in 1x TAE buffer and stained with ethidium bromide and documented by gel doc system.

### Anchor-primer ligation of dsRNA

The obtained dsRNA was resolved on 1.5%agarose gel and the dsRNA segments were excised. DsRNA molecules that were close to each other were excised together as a mixed population. These dsRNAs were further purified by gel elution kit (Macherey Nagel, Germany). 200–300 ng of dsRNA was used for self- priming anchor primer ligation in a 60 μl reaction with T4 RNA ligase (Fermentas, Thermo Scientific, USA). Self-priming oligo (5′p-GACCTCTGAGGATTCTAAAC/iSp9/TCCAGTTTAGAATCC-OH 3′), having C9 internal spacer region (iSp9) was used for ligation at 3′end -OH group of dsRNA [89]. The ligation buffer [90] was modified to contain 50 mM HEPES/NaOH buffer pH 8.0, 15 mM MgCl_2_, 0.01% BSA, 0.75 mM ATP (Fermentas, Thermo Scientific, USA), 1.5 mM DTT (Fermentas, Thermo Scientific, USA), 8% DMSO (Sigma, USA), 15% polyethylene glycol 6000 (PEG_6000_) (Himedia, Mumbai, India) and 10 U T4 RNA ligase (Fermentas, Thermo Scientific, USA). Ligation was performed at 37°C for 12–16 hrs.

### Full-length cDNA synthesis and PCR amplification

Ligated dsRNA was purified using gel extraction columns (Macherey-Nagel, Germany) in 15 μl volume. About 50–70 ng of ligated dsRNA were denatured in the presence of 1.5% DMSO and 2M betaine at 98°C for 2 min (optimal) and immediately chilled on ice. The denatured dsRNA was used for the first strand cDNA synthesis by reverse transcription (RT) reaction. The RT reaction mixture consisted of 1X RT buffer, 1 μl dNTPs (10 mM each), 1 μl (200 units) of RTase (Primescript, TakaraBio, Japan) and the final volume was adjusted to 25 μl with nuclease free water. The RT reaction mixture was incubated at 42°C for 1 hr and then 70°C for 15 min to deactivate the enzyme. The cDNA obtained was amplified by a primer (5′- GAGGGATCCAGTTTAGAATCCTCAGAGGTC-3′) complementary to anchor sequence [89]. Briefly, PCR reaction contained 25 μl reaction mixture [1.5 μl of first strand cDNA, 1.0 μl of 2.5 mM dNTPs mix, 1X GC melt buffer, 0.5 μl of 10 pmole primer and 0.2 μl (1unit) GC LA Taq (TakaraBio, Japan)]. Amplification cycle was: 94°C for 1min followed by 35 cycles of denaturation at 94°C for 30 s, annealing at 62°C for 30 s and extension at 72°C for 2.30 min. Final extension was carried for 72°C for 5 min. RT-PCR amplicons were resolved on 1% agarose gel containing ethidium bromide and visualized in gel doc.

### Cloning and sequencing

Amplicons corresponding to the dsRNA bands were excised from gel, purified using GenElute Gel Extraction kit (Sigma–Aldrich, USA) and cloned into pGEMT-easy vector (Promega, Madison, Wisconsin, USA). Recombinant plasmids were purified using GenElute Plasmid Miniprep kit (Sigma–Aldrich, USA) and the target cDNAs were sequenced in an automated DNA sequencer (ABIPRISM^®^3130xl Genetic Analyzer) using ABI prism Big Dye^™^ Terminator v3.1 Ready Reaction Cycle Sequencing kit (Applied Biosystems, USA), with 2X coverage. Nucleotide sequences were translated using Expasy server (http://web.expasy.org/translate), and the obtained amino acid sequence was used to determine homology by Blastp (http://www.ncbi.nlm.nih.gov/blast) analysis.

### Computer analysis of the ArCV-1 3D structure of RdRp

We studied the three dimensional crystal structure of RdRp using computer aided analysis. The amino acid sequence of ArCV-1 (Table 1), RdRp (P1), was used to develop 3D structure with characteristic folding. I-TASSER (http://zhanglab.ccmb.med.umich.edu/I-TASSER), an online server was used to study the RdRp [37, 38]. This integrated platform generated three-dimensional atomic models from multiple threading alignments and iterative structural assembly simulations. Data provided on topology similarity (TM) with the proteins structurally close to the target protein (s) in the PDB is helpful contrivance to assess the relationships of the polymerase. The outputs of the I-TASSER data of possible predicted models and the molecular graphics are of high quality that contained full-length secondary and tertiary structure predictions. Developed structures were subjected to simulations by an open-source viewer, the PyMol (https://www.pymol.org) and Jmol (http://jmol.sourceforge.net/) an interactive graphics program [91, 92], for illustrating the three-dimensional (3D) chemical structures of the crystal. These programs were used to alter the scheme of the images to characterize the proteins. Using this program, individual and conserved residues in the motifs were identified which facilitated understanding over all nature of the RdRp. Structural adjustment was made for this model of ArCV-1 3D ^pol^ of about 90° to study from top surface and side ways to visualize the orientation of channels. Amino acid sequences of 3Dpol structures of Norwalk virus (1SH0), poliovirus (4R0E) and Sapporo virus (2wk4A) from PDB and RTSV sequence (NCBI NP734463) was used in the analysis and to develop 3Dpol structures.

### Construction of phylogenetic trees

Multiple alignments and sequence identity matrix of RdRp and dsRNA capsid protein-like sequences were carried out by ClustalW [93]. To deduce the evolutionary relationship, the phylogenetic tree was prepared by MEGA 6.0 program [94], by using neighbor-joining method [95], and evolutionary distances were computed using the maximum composite likelihood method with 1000 bootstrap value [96]. The bar represents base substitution per site. Sequence identity matrix and phylogeny were studied by using NCBI GenBank database (http://www.ncbi.nlm.nih.gov/genbank).

## Supporting information

S1 Fig Structural alignment of conserved amino acid residues of putative RdRp (RNA-1) of some members of genus *Deltapartitivirus* with ArCV-1. (DOCX)

S2 Fig Different motifs and individual amino acid positions in ArCV-1, 3D pol. (DOCX)

S3 Fig 3D pol structural similarities of cryptoviruses, plant and human picornaviruses. (DOCX)

S1 Table Details of different partitiviruses sequence retrieved from NCBI database, used in the phylogenetic analysis. (DOCX)
