# Supplementary material for: Molecular characterization of a novel cryptic virus infecting pigeonpea plants
Source: PLoS One. 2017 Aug 3;12(8):e0181829. doi: 10.1371/journal.pone.0181829 (PMC5542627; doi:10.1371/journal.pone.0181829)
Supplement: S1 Fig — (DOCX) [file pone.0181829.s001.docx]

**
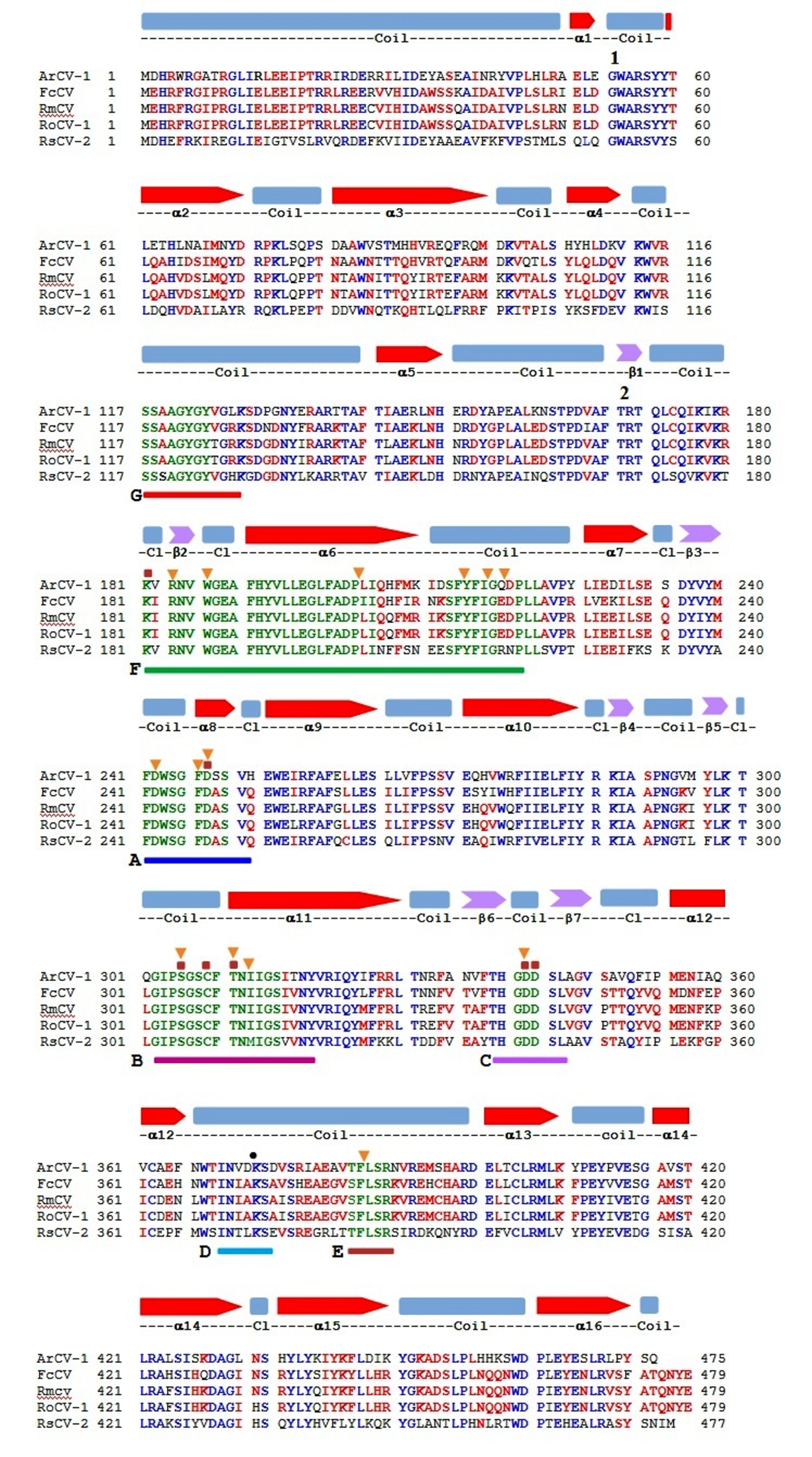
Supporting Information:**

**S1 Fig.** **Structural alignment of conserved amino acid residues of putative RdRp (RNA-1) of some members of genus *Deltapartitivirus* with ArCV-1.** *Arhar cryptic virus*-1 (ArCV-1; HG797710), *Fragaria chiloensis cryptic virus* (FcCV; DQ093961), *Rosa multiflora cryptic virus* (RmCV; EU024675), *Rose cryptic virus-1* (RoCV-1; EU413666) and *Raphanus sativus cryptic virus*-2 (RsCV-2; DQ218036) are the tripartite cryptoviruses used in the study. A to F and G motifs mostly universal with conserved active residues are identified and represented by color coded bars. Conserved residues in a column are highlighted in blue. Three or more residues which are identical were colored in red. The active residues of the sub domains are denoted by orange triangles and the motifs colored by green. Residues predicted to be interacting with the incoming NTP substrate was identified in ArCV-1 RdRp, are marked by brown squares. The predicted secondary structure from the peptide sequence as the random coils (solid light blue) α-helices (red arrow) and β-strands (magenta chevron) were aligned above with the ArCV-1 sequence.
