# Supplementary material for: Molecular characterization of a novel cryptic virus infecting pigeonpea plants
Source: PLoS One. 2017 Aug 3;12(8):e0181829. doi: 10.1371/journal.pone.0181829 (PMC5542627; doi:10.1371/journal.pone.0181829)
Supplement: S2 Fig — (DOCX) [file pone.0181829.s002.docx]

**
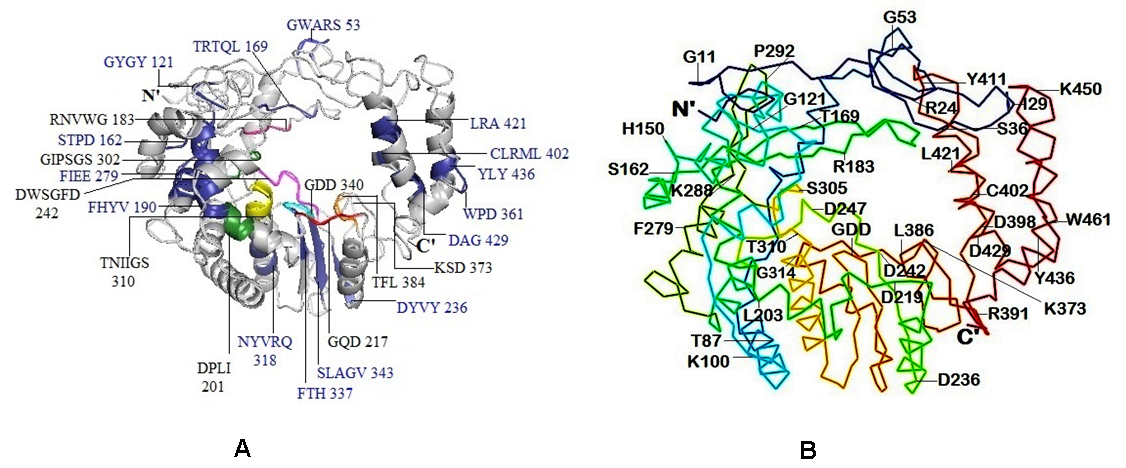
Supporting information:**

**S2 Fig. Different motifs and individual amino acid positions in ArCV-1, 3D pol. (A)** ArCV-1 3D ^pol^ in cartoon showing conserved amino acids in each of the several motifs is indicated. **(B)** Stereo view backbone representation of the ArCV-1 RdRp showing the amino acid positions relative to the secondary structure and the position of amino acids indicated from the N-terminus (blue) to C-terminus (red) of the polymerase.
