# Supplementary material for: Molecular characterization of a novel cryptic virus infecting pigeonpea plants
Source: PLoS One. 2017 Aug 3;12(8):e0181829. doi: 10.1371/journal.pone.0181829 (PMC5542627; doi:10.1371/journal.pone.0181829)
Supplement: S3 Fig — (DOCX) [file pone.0181829.s003.docx]

**Supporting information:**


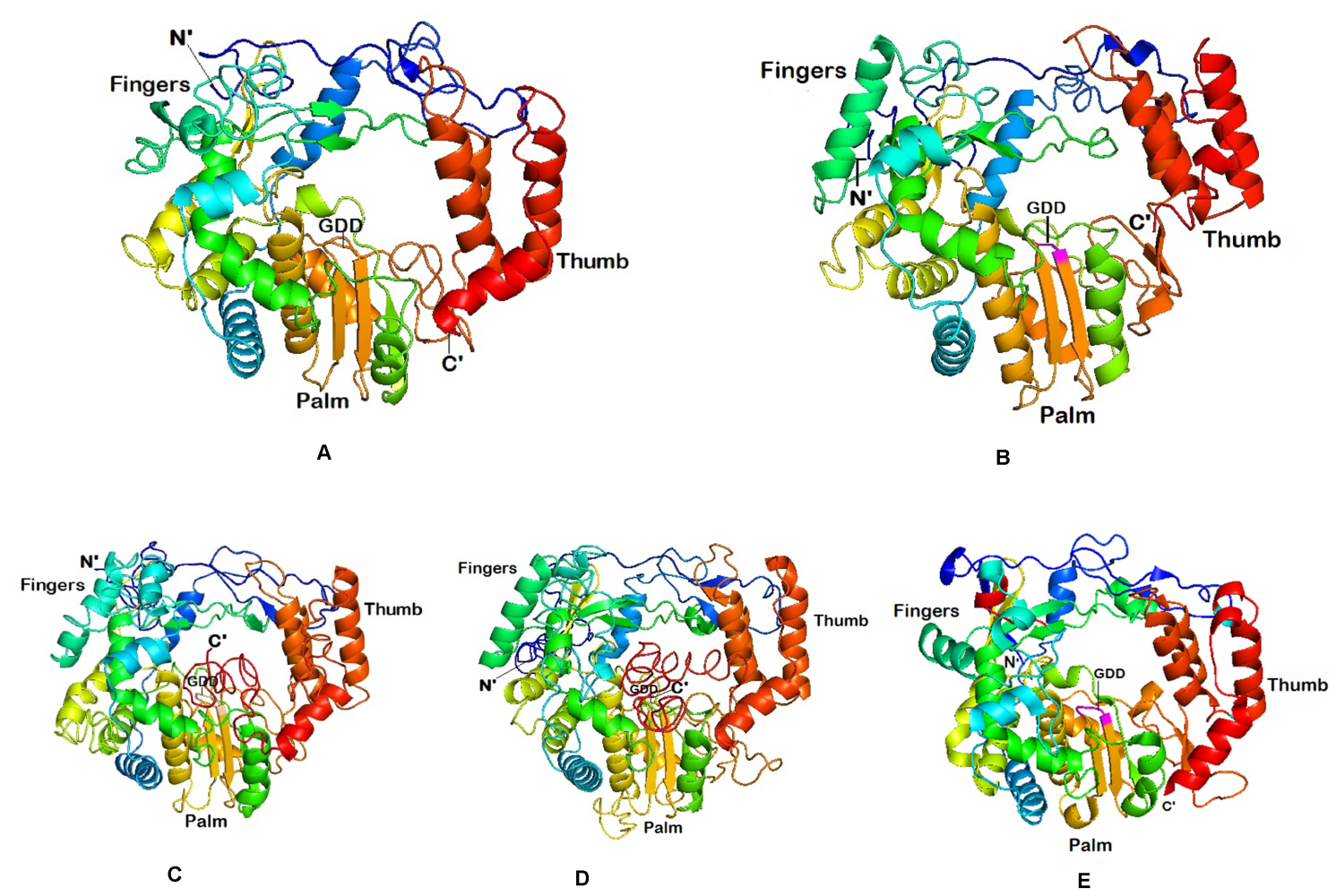


**S3 Fig. 3D pol structural similarities of cryptoviruses, plant and human picornaviruses.** The cryptovirus and picornavirus RdRps showing over all structural similarity. **(A)** 3Dpol cartoon representation of Arhar cryptic virus*-*1 (ArCV-1; Tripartite Deltapartitivirus, NC 024014). **(B)** Cartoon representation of Raphanus sativus cryptic virus*-*3 (RsCV-3; Bipartite *Deltapartitivirus,* NC 011705). **(C)** Cartoon representation of Raphanus sativus cryptic virus*-*1 (RsCV-1; Tripartite *Alphapartitivirus,* NC 008191). **(D)** 3Dpol of Rice tungro spherical virus (RTSV, Linear ssRNA (+) genus *Waikavirus*, *Picornavirales,* NP 734463) showing C-terminus end close to the catalytic center (GDD) in the central cavity similar to RsCV-1and Norwalk virus (Fig. 5D) and **(E)** RdRp of Poliovirus (PV, Linear ssRNA (+) genus *Enterovirus* *Picornaviridae* family, PDB-4R0E), infecting humans.
