# Supplementary material for: Molecular characterization of a novel cryptic virus infecting pigeonpea plants
Source: PLoS One. 2017 Aug 3;12(8):e0181829. doi: 10.1371/journal.pone.0181829 (PMC5542627; doi:10.1371/journal.pone.0181829)
Supplement: S1 Table — (DOCX) [file pone.0181829.s004.docx]

**S1 Table.** **Details of different partitiviruses sequence retrieved from NCBI database, used in the phylogenetic analysis.**

| **Genus** | **Virus** | **Gene bank accession no. for** | | |
| --- | --- | --- | --- | --- |
|  |  | RNA-1 | RNA-2 | RNA-3 |
| ***Alphapartitivirus*** | Beet cryptic virus 1 (BCV-1) | EU489062 | EU489062 |  |
|  | Raphanus sativus cryptic virus1(RsCV-1) | AY949985 | DQ181926 | DQ181927 |
|  | Red clover cryptic virus 1 (RCCV-1) | KF484724 | KF484725 |  |
|  | Vicia cryptic virus (VCV) | EF173396 | EF173395 |  |
|  | White clover cryptic virus 1 (WCCV-1) **TS*** | AY705784 | AY705785 |  |
|  |  |  |  |  |
| ***Betapartitivirus*** | Atkinsonella hypoxylon virus (AhV) **TS** | NC_003470 | NC_003471 | NC_003472 |
|  | Cannabis cryptic virus (CanCV) | JN196536 | JN196537 |  |
|  | Crimson clover cryptic virus 2 (CCCV-2) | JX971982 | JX971983 |  |
|  | Fusarium poe virus 1 (FpV-1) | AF047013 | AF015924 |  |
|  | Hop trefoil cryptic virus 2 (HTCV-2) | JX971980 | JX971981 |  |
|  | Red clover cryptic virus 2 (RCCV-2) | JX971978 | JX971979 |  |
|  | White clover cryptic virus 2 (WCCV-2) | JX971976 | JX971977 |  |
|  |  |  |  |  |
| ***Deltapartitivirus*** | Beet cryptic virus 2 (BCV-2*)* | HM560702 | HM560703 | HM560704 |
|  | Beet cryptic virus 3 (BCV-3) | S63913 |  |  |
|  | Fig cryptic virus (FCV) | FR687854 | FR687855 |  |
|  | Fragaria chiloensis cryptic virus (FcCV) | DQ093961 | DQ355440 | DQ355439 |
|  | Pepper cryptic virus 1 (PepCV-1) **TS** | JN117276 | JN117277 |  |
|  | Pepper cryptic virus 2 (PepCV-2) | JN117278 | JN117279 |  |
|  | Raphanus sativus cryptic virus 2 (RsCV-2) | DQ218036 | DQ218037 | DQ218038 |
|  | Raphanus sativus cryptic virus 3(RsCV-3) | FJ461349 | FJ461350 |  |
|  | Rose cryptic virus 1 ShB-1 (RoCV-1) | EU413666 | EU413667 | EU413668 |
|  | Rose cryptic virus 1 (RoCV-1) | NC_010346 | NC_010347 | NC_010348 |
|  | Rosa multiflora cryptic virus (RmCV) | EU024675 | EU024676 | EU024677 |
|  |  |  |  |  |
| ***Gammapartitivirus*** | Aspergillus ochraceous virus (AoV) | EU118277 | EU118278 | EU118279 |
|  | Ophiostoma partivirus 1 (OpV-1) | AM087202 | AM087203 |  |
|  | [Penicillium stoloniferum virus S](http://en.wikipedia.org/w/index.php?title=Penicillium_stoloniferum_virus_S&action=edit&redlink=1) (PsV-S**)TS** | NC_005976 | NC_005977 |  |
|  |  |  |  |  |
| ***Cryspovirus*** | Cryptosporidium parvum virus 1 (CSpV-1) **TS** | U95995 | U95996 |  |
|  |  |  |  |  |
| **Unclassified** | Carrot cryptic virus (CCV) | FJ550604 | FJ550605 |  |
|  | Ceratocystis polonica partivirus (CpPV) | AY260756 | AY260757 |  |
|  | Citrullus lanatus cryptic virus (CiLCV) | KC429582 | KC429583 |  |
|  | Mulberry cryptic virus 1(MbCV-1) | ACZ54927 | ACZ54928 |  |
|  | Mycovirus fusarium solani virus (MFusoV) | D55668 | D55669 |  |
|  | Persimmon cryptic virus (PerCV) | HE805113 | HE805114 |  |

TS= Type species
